# Form (III) of artemisinin: discovery and crystallographic characterization of a new high-pressure polymorph

**DOI:** 10.1107/S205252062600291X

**Published:** 2026-05-11

**Authors:** Banaz Fetah, Lauren E. Connor, Mark R. Warren, Cheryl L. Doherty, Iain D. H. Oswald

**Affiliations:** a Strathclyde Institute of Pharmacy & Biomedical Sciences (SIPBS) University of Strathclyde 161 Cathedral Street Glasgow G4 0RE UK; b Biomedical Research, Novartis, Basel, 4002, Switzerland; c Diamond Light Source, Harwell Science and Innovation Campus, Chilton, Didcot, OX11 0DE, UK; d Material Science GlaxoSmithKline Gunnels Wood Rd Stevenage SG1 2NY UK; UPES University of Tomorrow, India

**Keywords:** diamond anvil cell (DAC), high pressure, polymorphism, single crystal, XRPD, artemisinin, compression-induced transformation

## Abstract

Artemisinin (ART) is mainly used for the treatment of malaria and exhibits polymorphism with two known crystalline forms. In this study, the high-pressure behaviour of these two polymorphs was investigated to evaluate their compressibility and identify if any pressure-induced phase transitions occur with a view to assessing the impact of manufacturing pressure on the active pharmaceutical ingredient. Form (I), the orthorhombic polymorph, is found to be the most compressible of the three. Form (II), a triclinic phase, undergoes a phase transition to a new polymorph that is observed at different pressures depending on the pressure-transmitting medium (PTM) used. The transition to form (III) occurs at 0.75 GPa when compressed in petroleum ether, however, this transition is delayed to 2.02 GPa in silicone oil. This highlights the influence of the PTM on the stability of the crystal structure. The newly characterized form (III) shares structural similarities with form (II) but differs in symmetry where a pseudo-2_1_ screw axis in form (II) becomes a formal 2_1_ screw axis in form (III), resulting in a change from triclinic to monoclinic and a reduction of the asymmetric unit from *Z*′ = 4 to *Z*′ = 2. These findings contribute to a deeper understanding of pressure-induced polymorphism in ART and underscore the importance of external factors, such as PTM, in influencing solid-state transitions relevant to pharmaceutical processing and formulation.

## Abstract 2

The polymorphic behaviour of the three forms of artemisinin was investigated using single-crystal X-ray diffraction in a diamond anvil cell, with notable observation of a pressure-induced phase transition from form (II) to form (III).

## 1. Introduction

It is common to formulate active pharmaceutical ingredients (APIs) in the solid state for oral administration due to the non-invasive nature of this delivery route, stability of API and relative ease of production (Alqahtani *et al.*, 2021 ▸). The identification of a solid form of the API with suitable properties and behaviours for this route of delivery is essential in the development of a successful drug product (Ticehurst & Marziano, 2015 ▸). Polymorphism, the ability of a molecule to exist in more than one crystalline form, can impact these physical properties by altering the free energy of the solid through changes in the molecular packing and intermolecular interactions (Bellur Atici & Karlığa, 2015 ▸; Bernstein, 2011 ▸). These changes impact the physicochemical properties of the polymorph when compared with the original form, potentially altering solubility and mechanical properties and impacting its processibility and efficacy (Censi & Di Martino, 2015 ▸). Therefore, if two polymorphs of the same compound exhibit different solubilities, then the bioavailability of the polymorphs may vary, which can have significant consequences for the accurate administration of the appropriate dosage to the patient. The antibiotic chloramphenicol palmitate is a good example to highlight the impact of polymorphism on bioavailability. The polymorphic forms of chloramphenicol palmitate include polymorph A (stable), polymorph B (metastable) (Eguchi & Iitaka, 1974 ▸) and polymorph C (unstable). Gamberini *et al.* (2006 ▸) used a combination of X-ray diffraction, spectroscopic techniques and thermal methods to differentiate between the three different forms. The authors were able to use specific wavelength ranges in the Raman spectra (413–435 and 1035–1158 cm^−1^) to establish a linear relationship between the quantity of polymorph A in 2–14% mixtures of A and B polymorphs. Polymorph A is the thermodynamically stable form and is the inactive form of chloramphenicol palmitate, whilst polymorph B is the active form and is much more soluble, leading to an increase in dissolution rate. Aguiar *et al.* (1967 ▸) found a significant increase in the peak serum levels if form B was used compared with form A (22 µg ml^−1^ for form B compared with 3 µg ml^−1^ for form A). This is a significant change in the bioavailability of chloramphenicol palmitate and highlights the importance of control of the polymorphic forms. The extent of polymorphism in pharmaceutical materials has been captured by Cruz-Cabeza *et al.* (2015 ▸), showing that more than 50% of small organic molecules exhibit polymorphism. Cruz-Cabeza *et al.* (2015 ▸) investigated the difference in observed polymorphism in the Cambridge Structural Database (CSD) with respect to those found in industry databases. They found that polymorphism occurs in 37% of single-component materials in the CSD, rising to 53% and 66% in the Roche and Lilly databases, respectively. This increase is at least partly due to the pharmaceutical companies specifically conducting polymorphic screening during the early stages of drug development as it is critical to identify all possible solid forms to ensure drug stability and to facilitate the development of drug products to the satifaction of regulatory agencies. These agencies require strict controls on solid form across batches to ensure consistency of action and a detailed understanding of the solid form landscape under processing and manufacturing conditions (Kestur *et al.*, 2023 ▸).

An area of increased research activity is the monitoring of polymorphism during tabletting. Under tabletting conditions, powders can experience pressures up to 300 MPa, which has been shown to promote transitions to other polymorphs during processing (Thakral *et al.*, 2019 ▸; Park *et al.*, 2022 ▸; Wildfong *et al.*, 2007 ▸; Rogers *et al.*, 2013 ▸). Analysis of tabletted forms was explored in chloramphenicol palmitate by Lin *et al.* (2006 ▸), who used it as a test subject to explore multivariate methods on Raman data for polymorphic identification in both a free powder and a processed tabletted form. Whilst this method development study investigated pre-set mixtures of polymorphs, other studies have indicated that changes to polymorphic forms under compression can occur. Form B of famotidine has been shown to be unstable to compression with conversion to form A (Roux *et al.*, 2002 ▸; Német *et al.*, 2005 ▸; Lin, 2014 ▸; Upadhyay *et al.*, 2022 ▸). Otsuka *et al.*, (1989 ▸) and Thakral *et al.* (2019 ▸) investigated polymorphic transitions in chlorpropamide on tabletting. Thakral *et al.*, (2019 ▸) showed that conversion between form A (α) to form C (γ) occurs and is dependent on the position within the tablet. The percentage conversion was observed to be higher at the edges of the tablet than in the centre. The spatial dependence suggests a link to localized variations in pressure during compaction; however, the impact of heat cannot be ruled out as a route of polymorphic conversion (Zavaliangos *et al.*, 2008 ▸). In fact, Boldyreva *et al.* (2006 ▸) did not observe the transition in chlorpropamide in a diamond anvil cell (DAC), stating that local heating effects are likely to be the cause of the transition. The previous work involved the destructive analysis of discrete tablet sections. In contrast, Gasol-Cardona *et al.* (2025*b* ▸) employed X-ray diffraction computed tomography to analyse glycolide tablets non-destructively, detecting the presence of a high-pressure phase at compaction pressures as low as 250 MPa. Furthermore, work by Kakde *et al.* (2025 ▸) explored the use of DACs as part of a polymorph screening protocol. They investigated hydro­chloro­thia­zide using a combination of texture analyser (TA) and DAC techniques to reveal that phase transitions occurring in the DAC at 500 MPa also appeared under tabletting conditions in the TA at 300 MPa. Collectively, the materials used in the latter studies have been investigated using both DAC (to reveal structure) and manufacturing processes (to reveal the impact of the process) and highlights the potential of DAC studies to simulate pressure-induced phase transitions relevant to pharmaceutical tabletting.

Building on these findings, this study focuses on the solid-state behaviour of a pharmaceutically relevant compound under high pressure. Artemisinin (ART) (see Scheme 1) is a sesquiterpene lactone with an endoperoxide 1,2,4-trioxane ring.
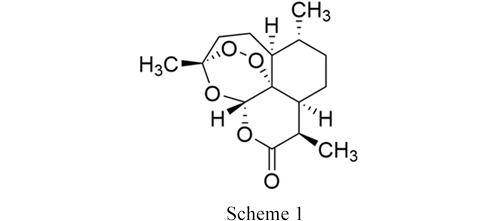
ART is used for treating malaria and derived from sweet wormwood extracts. ART exhibits two observable crystalline forms under ambient-temperature conditions; orthorhombic form (I) and triclinic form (II) which have been previously identified and their crystal structures determined (Lisgarten *et al.*, 1998 ▸; Chan *et al.*, 1997 ▸). Further co-crystals with orcinol and resorcinol have been identified via mechanochemical methods and their structures characterized (Karki *et al.*, 2010 ▸). The orthorhombic form is thermodynamically stable at low temperatures, whereas the triclinic form is stable at higher temperatures with the two polymorphs being enantiotropically related (Horosanskaia *et al.*, 2014 ▸). The phase transition was determined to be at 130°C using differential scanning calorimetry and temperature-resolved X-ray diffraction (Horosanskaia *et al.*, 2014 ▸). Form (II) is observed over the entire temperature range (to 147°C; melting point 153°C) and on cooling to ambient temperature showing its resistance to transformation. To date, the response of ART to pressure is unknown. Given that pressure plays a crucial role in manufacturing processes and that high-pressure polymorphs have been observed in tablets, we investigated the behaviour of the known polymorphs of ART under high-pressure conditions to evaluate its structural stability and determine whether a pressure-induced phase transition could be observed.

## 2. Materials and methods

### 2.1. Materials

ART was purchased from Alfa Aesar. The solvents used were ethanol (99.8%) purchased from Fisher and cyclo­hexane (≥99.0%) purchased from Sigma Aldrich.

### 2.2. Method of preparation for recrystallization

To obtain crystals of form (II) of ART, ART was slurried in cyclo­hexane (1 g ml^−1^) using two magnetic stirrers at 340 rpm and ∼293 K for 24 h. To obtain single crystals for analysis, the supernatant solution was filtered through a pre-soaked 0.2 µm PTFE filter into a different glass vial. The glass vial was covered with aluminium foil and perforated for slow evaporation of the solvent at ∼293 K to obtain single crystals.

### 2.3. High-pressure DAC

A Merrill–Bassett DAC with a half-opening angle of 40° was used to apply pressure to single crystals of ART. The two diamonds with 600 µm culets were secured in tungsten carbide backing seats. A 250 µm-thick gasket, which functions as the sample chamber for the DAC, was drilled with a 200 µm or 300 µm tungsten carbide drill using the Almax Easylab Driller. A ruby chip was added to the sample chamber along with petroleum ether [pressure-transmitting medium (PTM)] for hydro­static compression. The pressure was determined using the ruby fluorescence technique (Shen *et al.*, 2020 ▸; Forman *et al.*, 1972 ▸).

#### 2.3.1. High-pressure annealing

A similar set-up was used for the annealing process to grow better crystals of the triclinic phase. In this case, the supersaturated cyclo­hexane solution of ART was used as solvent to anneal the crystal. The DAC was heat cycled around ∼130°C to form the single crystal. After cooling, the crystal was subsequently loaded into a separate DAC and petroleum ether was used as the PTM to perform the compression study.

### 2.4. Sapphire capillary cell (SCC)

Data were collected using the high-pressure sapphire capillary cell for single crystals from 30 bar (0.003 GPa) up to 1200 bar (0.12 GPa) in Hutch 2 on beamline I19 at Diamond Light Source (McMonagle *et al.*, 2020 ▸). Radiation of wavelength 0.48590 Å was used, with beam size of 100 µm × 100 µm in both horizontal and vertical directions. Data collection employed a Newport four-circle kappa goniometer and a DECTRIS Eiger2 4M CdTe detector. Crystal centering and acquisition was controlled using DLS’s in-house *GDA* (Diamond Light Source, 2003 ▸) software. Data were collected following a standard strategy optimized for the sapphire capillary cell using a φ scan from −170 to 175° at a step size of 0.2° for 0.2 s exposure. Reduction was performed using *xia2* (Winter, 2010 ▸) with *DIALS* (Diffraction Integration for Advanced Light Sources; Waterman *et al.*, 2013 ▸) and *CrysAlisPro* (Rigaku Oxford Diffraction, 2015 ▸) to achieve optimal integrated intensities. This process included integration, absorption correction and space-group determination. Samples were measured across a pressure range of 0.002 GPa to 0.12 GPa).

The original sapphire capillary cell method involved using a carbon fibre tube; however, this method was altered to use thin Kapton film with small notches cut out of the film to aid the centring of the sample in the cell due to the poor visualization of the crystal in the cell (Gasol-Cardona *et al.*, 2025*a* ▸). Single crystals of ART in cyclo­hexane were loaded onto the Kapton film near the notches to aid alignment.

### 2.5. Single-crystal X-ray diffraction (SC-XRD)

A Bruker D8 Venture diffractometer (Cu *K*α1, λ = 1.54043 Å) with a PHOTON II detector was used to collect at 296 K and its ambient pressure X-ray data. Data were indexed and integrated using *SAINT* (Bruker, 2016 ▸), which is included in the *APEX4* (Bruker, 2016 ▸) software. *SADABS* (Krause *et al.*, 2015 ▸) was used for applying the absorption correction. The crystal structure refinements were conducted with *SHELXL* (Sheldrick, 2015*a* ▸) via *Olex2-1.5* (Dolomanov *et al.*, 2009 ▸) using the coordinates of form (I), collected from the CSD (ref code: QNGHSU). The refined atomic coordinates at each pressure were used as the input for each of the following datasets.

High-pressure data were collected on two instruments. Firstly, a Bruker APEX-II diffractometer with Incoatec IμS microfocus X-ray source (Mo *K*α1, λ = 0.71073 Å) and a CCD detector was used for the collection of data for ART form (I). The data for the subsequent studies of ART form (II) and form (III) were collected on a Bruker D8 Venture diffractometer with IμS microfocus X-ray source (Mo *K*α1, λ = 0.71073 Å) and a PHOTON II detector. Data were indexed and integrated using *SAINT* (Bruker, 2016 ▸), which is included in *APEX4* (Bruker, 2016 ▸) software. *SADABS* (Krause *et al.*, 2015 ▸) was used for applying the absorption correction. The structures were refined in *Olex2-1.5* (Dolomanov *et al.*, 2009 ▸). Non-hydrogen atoms were assigned anisotropic displacement parameters. The Flack parameter refined as part of the crystal structure refinements are meaningless due to a combination of the radiation used and the atoms in the molecule. To access a larger portion of reciprocal space, Mo radiation was used; hence, the absolute structure could not be determined. A *Mogul* geometry check was carried out in *Mercury* for bond length, ring, and torsion angle abnormalities and valence angle and distance restraints were applied to the high-pressure structures based on the output. The triclinic dataset [form (II)] was refined using *CX-ASAP* (Thompson *et al.*, 2023 ▸). This program enables sequential refinement of crystal structures from a series of experimental data points (temperature/pressure) using *SHELXL* as the refinement package. The crystal structure refinement details for form (I) can be found in Table 1 ▸ and those of form (II) and (III) can be found in Table 2 ▸. The data points for the 0.75 GPa collection in petroleum ether and the 2.02 GPa collection in silicone oil were only sufficient to obtain unit-cell parameters. The data for these collections are found at the Zenodo website cited in the data availability statement.

### 2.6. Structural analysis

The equation of state (EoS) for the polymorphs was calculated using *EoSFit* (Angel *et al.*, 2014 ▸). The unit-cell parameters from forms (I) and (III) were used to fit a third-order Birch–Murnaghan EoS (BMEoS). A second-order BMEoS was used for form (II) due to the limited number of data points.

*ConQuest* was used to search for and retrieve structures listed in the Cambridge Structural Database (CSD; Groom *et al.*, 2016 ▸). The structures were visualized and analysed using *Mercury* (Macrae *et al.*, 2020 ▸). The *CellVol* (Wilson *et al.*, 2022 ▸) program was used to calculate the network and void volume of the systems as a function of pressure.

The *PIXEL* method was used to calculate intermolecular interaction energies of forms (I) and (III) facilitated by the *MrPixel* (Reeves *et al.*, 2020 ▸) script through *Mercury*. *Gaussian 09W* (Frisch *et al.*, 2009 ▸) was used to calculate the molecular electron density for input into *PixelC* (Gavezzotti, 2011 ▸; Gavezzotti, 2003 ▸). Both form (I) and form (III) possess *Z*′ ≤ 2, hence, the full *PixelC* calculation was performed to enable both the intermolecular interactions and the lattice energy to be calculated (Reeves *et al.*, 2020 ▸).

## 3. Results and discussion

### 3.1. Form (I)

**Figure 1:**
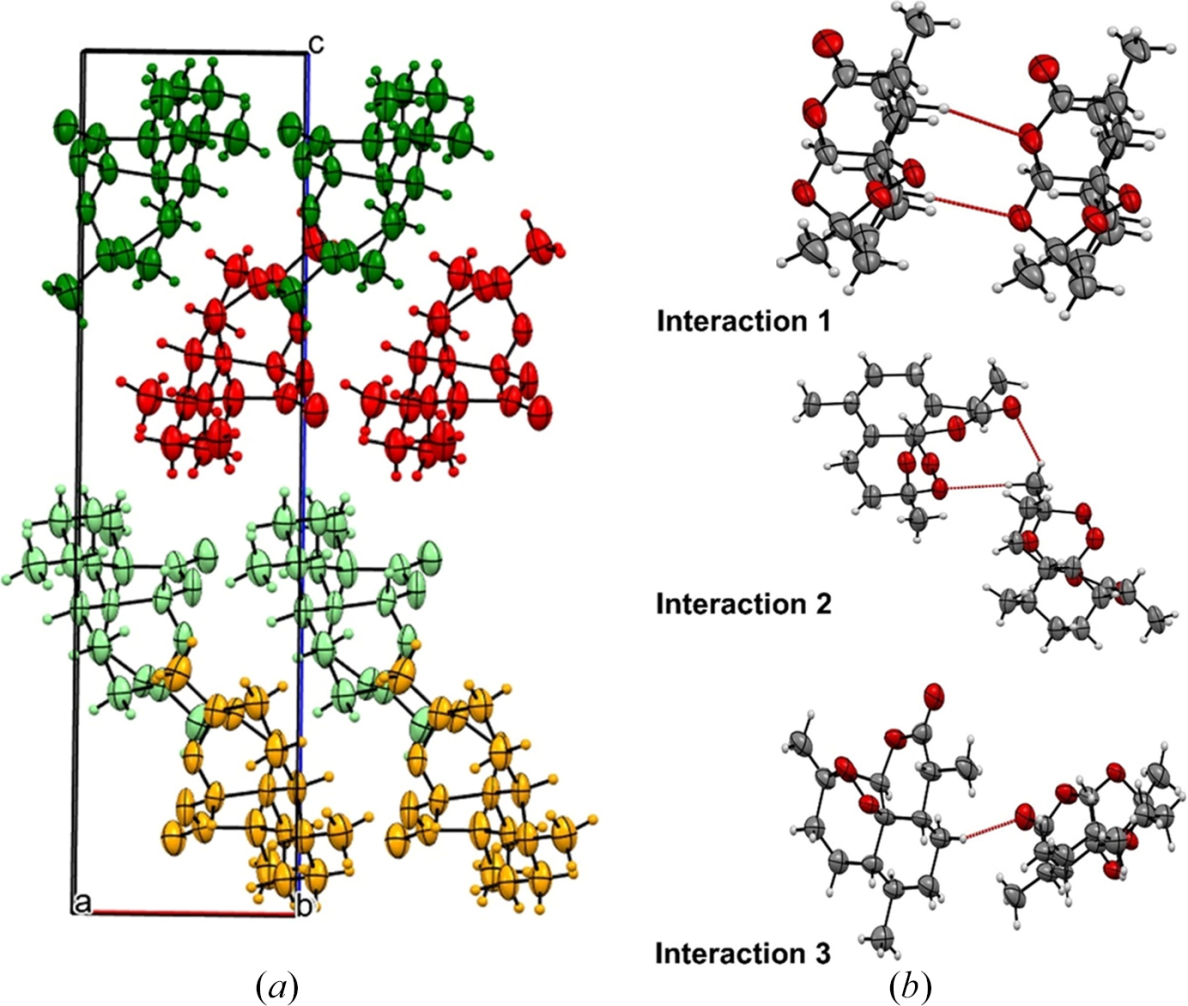
(*a*) Strongest interactions in the ART structure looking down the *b* axis. Interaction 1 (between molecules of similar colour), interaction 2 (orange and light-green) and interaction 3 (light-green to red). (*b*) Interactions 1–3 between pairs of molecules in the crystal structure.

To provide an overall understanding of form (I) we will firstly give a brief description of the ambient crystal structure before describing the changes as a function of pressure. Form (I) is the most stable polymorph of ART and crystallizes in orthorhombic *P*2_1_2_1_2_1_ with *Z*′ = 1. ART does not contain any hydrogen bond donors and so the crystal structure is composed of molecules interacting through weak CH⋯O and van der Waals interactions. The strongest interaction is between molecules stacked along the *a* direction involving a short contact between (C1—)H1⋯O3 [∼2.35 Å] and (C7—)H7⋯O4 [∼2.46 Å] [interaction 1; molecules of similar colour, −33.5 kJ mol^−1^; Fig. 1 ▸(*a*)]. Neighbouring chains are then related by the 2_1_-screw axis along the *b* direction (interaction 2; orange to light-green molecules or red to green molecules, −22.4 kJ mol^−1^; Fig. 1 ▸). The final interaction then links the molecules in the two halves of the unit cell together (interaction 3; −15.8 kJ mol^−1^; light-green to red molecules). The weaker interaction 3 results in a large portion of the void space being located in this region of the structure. Previous work has identified that void space and its compression can be a significant factor in the phase behaviour of a material (Turner *et al.*, 2011 ▸; Wood *et al.*, 2008 ▸; Wilson *et al.*, 2022 ▸; Ostrowska *et al.*, 2015 ▸).

#### 3.1.1. Effect of pressure on form (I)

ART  was investigated under two different pressure regimes: pressures from ambient to 1200 bar (0.12 GPa); and pressures from 0 to 5 GPa. The lower pressure regime was conducted using the sapphire capillary cell on beamline I19 at Diamond Light Source (McMonagle *et al.*, 2020 ▸).

ART compresses monotonically to 5 GPa without any change to the structure. The third-order BMEoS gives a bulk modulus (*K*_0_) of 2.9 (10) GPa, *V*_0_ = 1448.1 (10) Å^3^. As expected for a van der Waals solid, the material is relatively soft and its bulk modulus is lower compared with other van der Waals solids, *e.g.* naphthalene (*K*_0_ = 7.9 GPa) (Likhacheva *et al.*, 2014 ▸) or anthracene (*K*_0_ = 6.08 GPa) (Oehzelt *et al.*, 2002 ▸). Vaidya and Kennedy explored several other van der Waals compounds possessing bulk moduli exceeding that of ART, the lowest being biphenyl at *K*_0_ = 4.5 GPa (Vaidya & Kennedy, 1971 ▸). All of these examples involve planar molecules where the compression is largely between the planes. On the other hand, ART is non-planar, giving rise to compression in all directions. This added flexibility likely contributes to the material’s overall softness.

**Figure 2:**
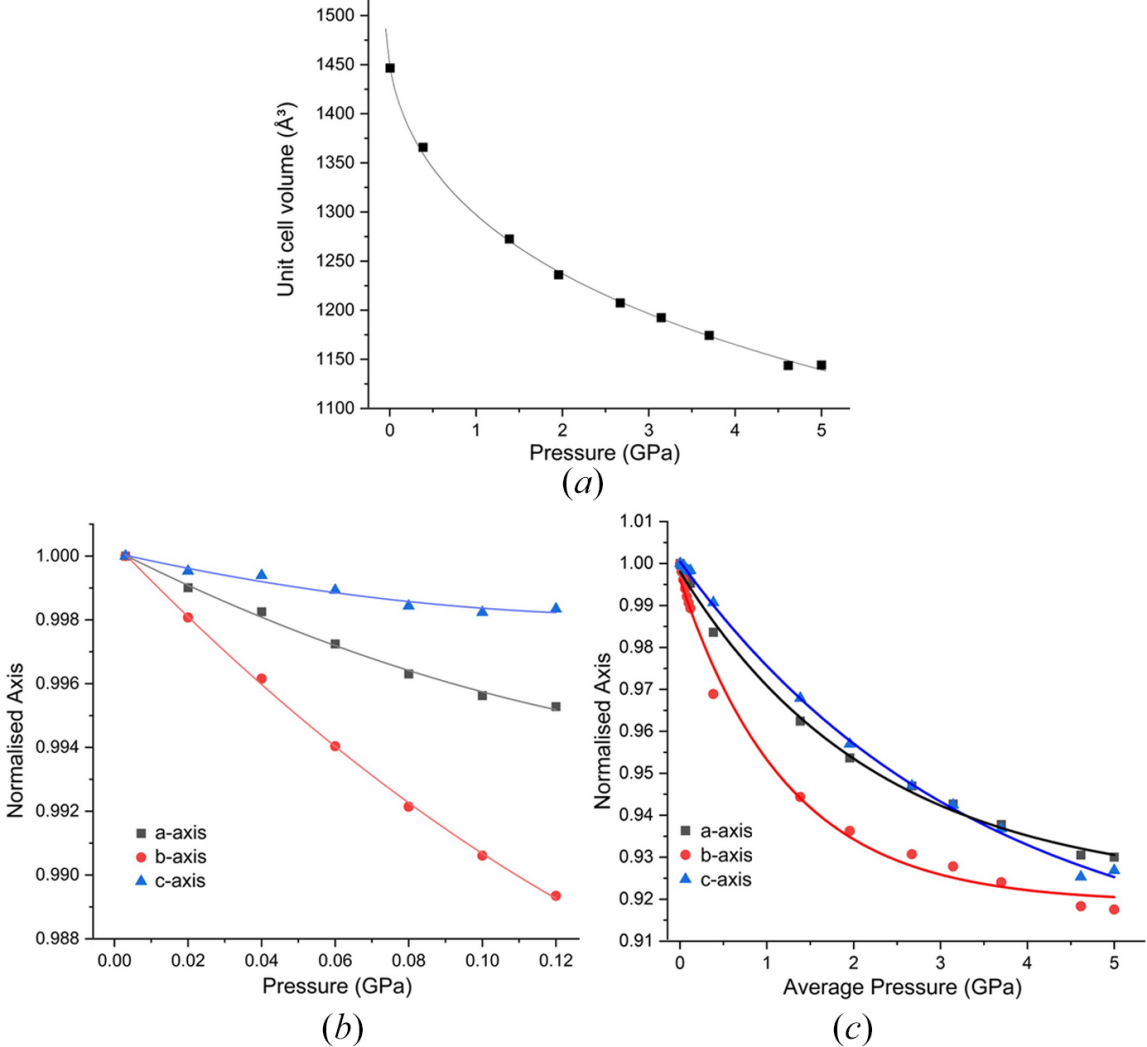
(*a*) Unit-cell volume fitted with third-order BMEoS as a function of pressure for form (I). (*b*) Compression behaviour of unit-cell parameters normalized using the ambient-pressure values in SCC. (*c*) Compression behaviour of unit-cell parameters from both SCC and DAC studies normalized using the ambient-pressure values in the DAC. Lines serve as a guide to the eye.

In response to hydro­static pressure, the structure of ART indicates that the biggest reduction occurs in the *b* axis (Fig. 2 ▸). SCC data [Fig. 2 ▸(*b*)] show a clearer distinction between the compressibility of all three axes, with the *b* axis still being the most compressible. The *a* and *c* axes initially show a different compressibility to each other but converge to a similar rate above ∼2 GPa.

**Figure 3:**
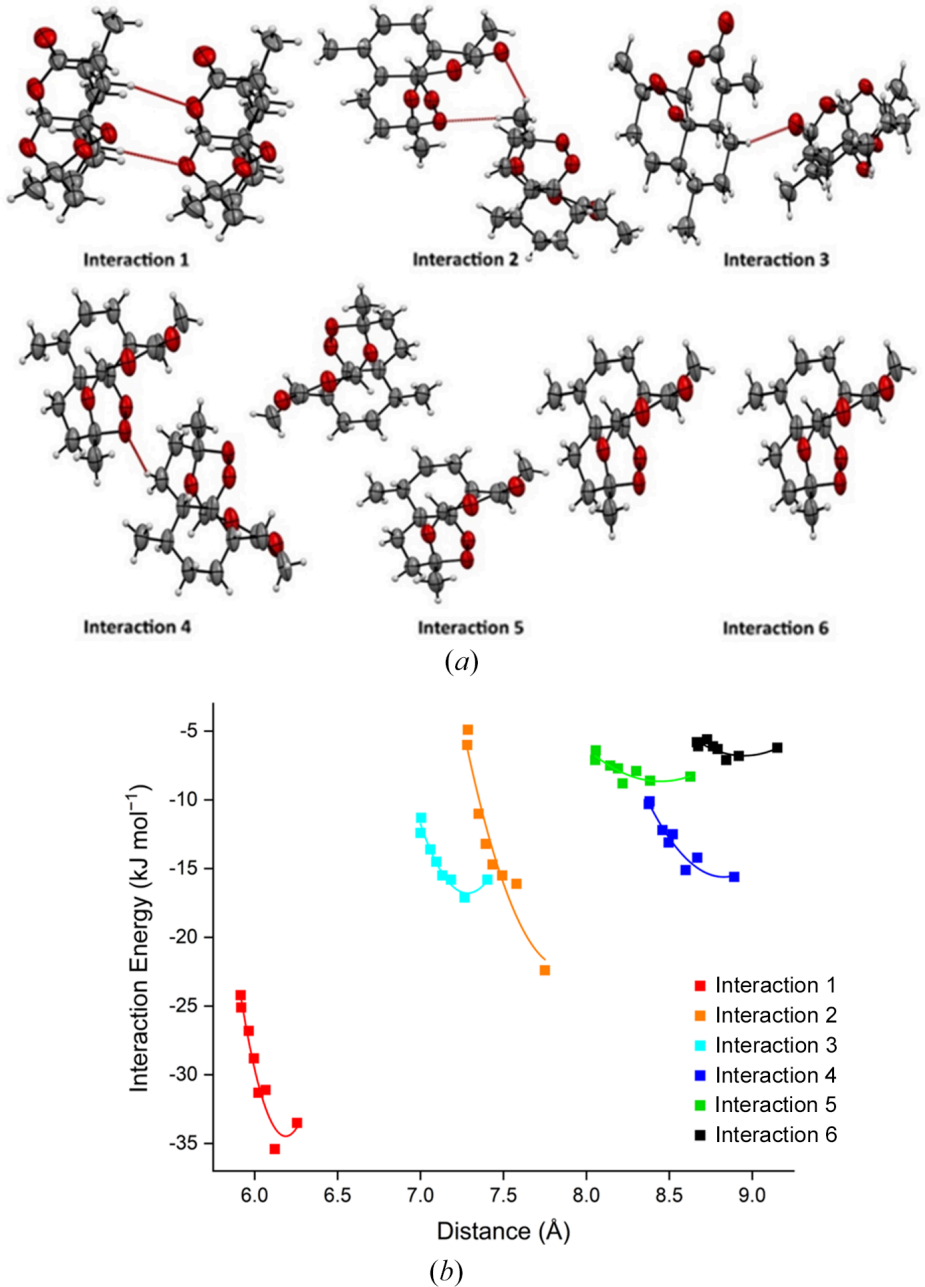
(*a*) Diagrams of the highest-energy interactions in the ART structure from PIXEL analysis. (*b*) Graph of the total interaction energy (in kJ mol^−1^) against the distance between the molecular centroids of the molecules involved in the interaction (in Å). Lines serve as a guide to the eye.

As the pressure increases, the overall lattice energy increases from −110.1 kJ mol^−1^ to −69.3 kJ mol^−1^ as a result of the steady relative increase in repulsion over attractive forces as molecules are pushed closer together. The internal molecular energy does not change substantially over the compression series (maximum 15 kJ mol^−1^). This suggests that the molecular conformation remains stable due to its rather rigid molecular structure, and that compression occurs through changes in intermolecular packing. We have followed the compression of the structure through the analysis of the six most energetically favourable pairwise interactions (labelled 1–6; Fig. 3 ▸). These interactions are the major contributors to the stabilization of the crystal structure. Interactions 1–3 display much steeper curves compared to interactions 4–6. The increase in energy penalty is primarily driven by repulsion as the molecules are compressed together (interaction 1: 30.9 to 104.8 kJ mol^−1^). Interaction 2 possesses a rather large centroid distance of ∼7.7 Å but still shows a steep increase in repulsion, which can be attributed to the orientation and proximity of a methyl group (C15) with respect to the oxygen atoms of the neighbouring molecule (O1 and O5). This leads to an increase in steric interactions and repulsion energy.

**Figure 4:**
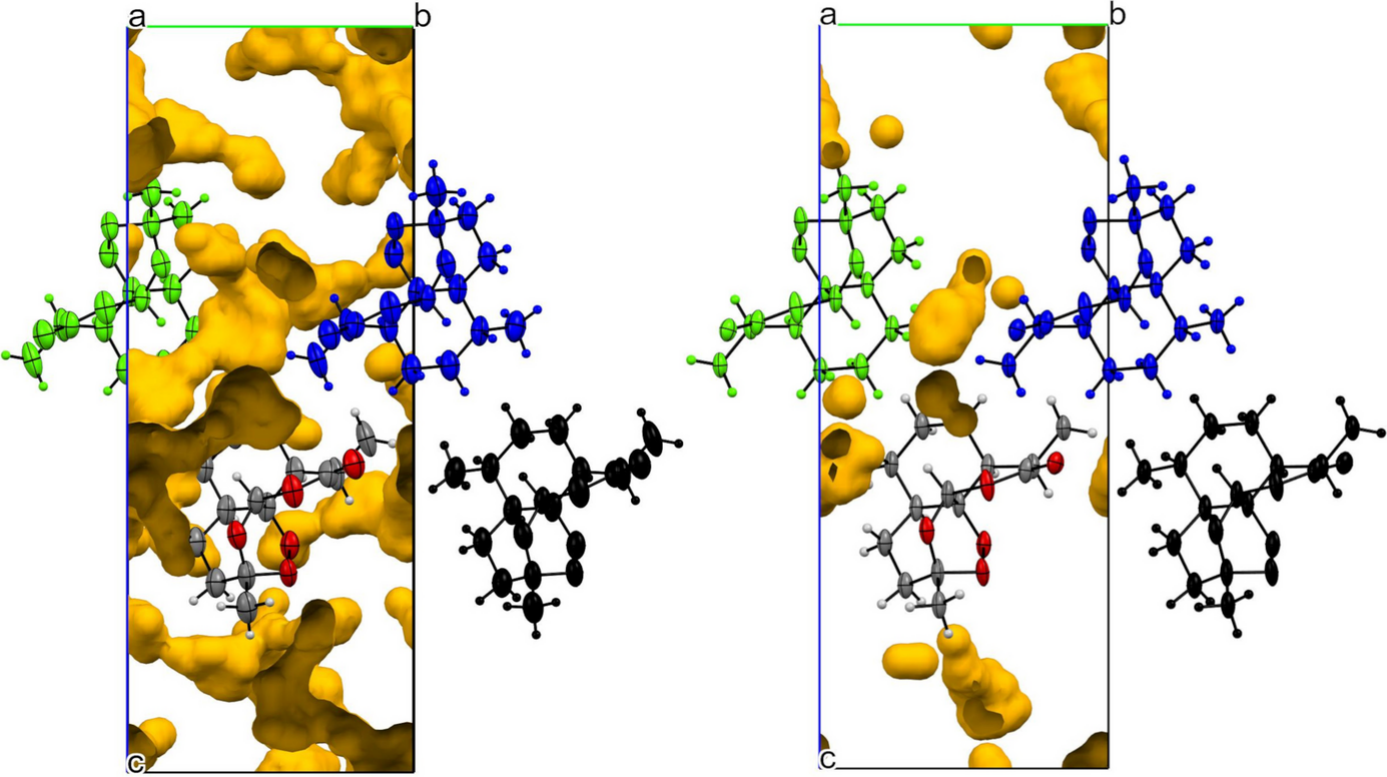
(*a*) Void spaces (yellow) in the ART form (I) structure at ambient pressure with interactions 4 (blue), 5 (green) and 6 (black) from the central molecule (atom colour). Void spaces at ambient pressure consist of 213.03 Å^3^ and 15.6% of the total unit-cell volume. (*b*) Void spaces in the ART structure at 5 GPa with the void spacing making up 43.72 Å^3^ and 3.8% of the unit cell. with same molecular colour scheme. Void space analysis was set with a probe of 0.5 Å radius and 0.2 Å grid spacing for both ambient pressure and 5 GPa.

The energies of interactions 4–6 are generally invariant on compression, suggesting that they are relatively soft. In particular, interactions 5 and 6 have a small energy penalty depicted by their flat curves in Fig. 3 ▸(*b*), and they can compress to a far greater extent than interaction 1. This behaviour can be rationalized by considering the distribution of void space within the crystal structure (Fig. 4 ▸). These voids, located around the central and lower regions of the unit cell, allow these molecules to move closer together and are visibly reduced at 5 GPa. These observations are commensurate with other studies that show that the location of the largest voids is often strongly correlated with the direction of greatest compressibility (Wood *et al.*, 2008 ▸; Tomkowiak & Katrusiak, 2019 ▸; Ward *et al.*, 2023 ▸).

### 3.2. Form (II)

**Figure 5:**
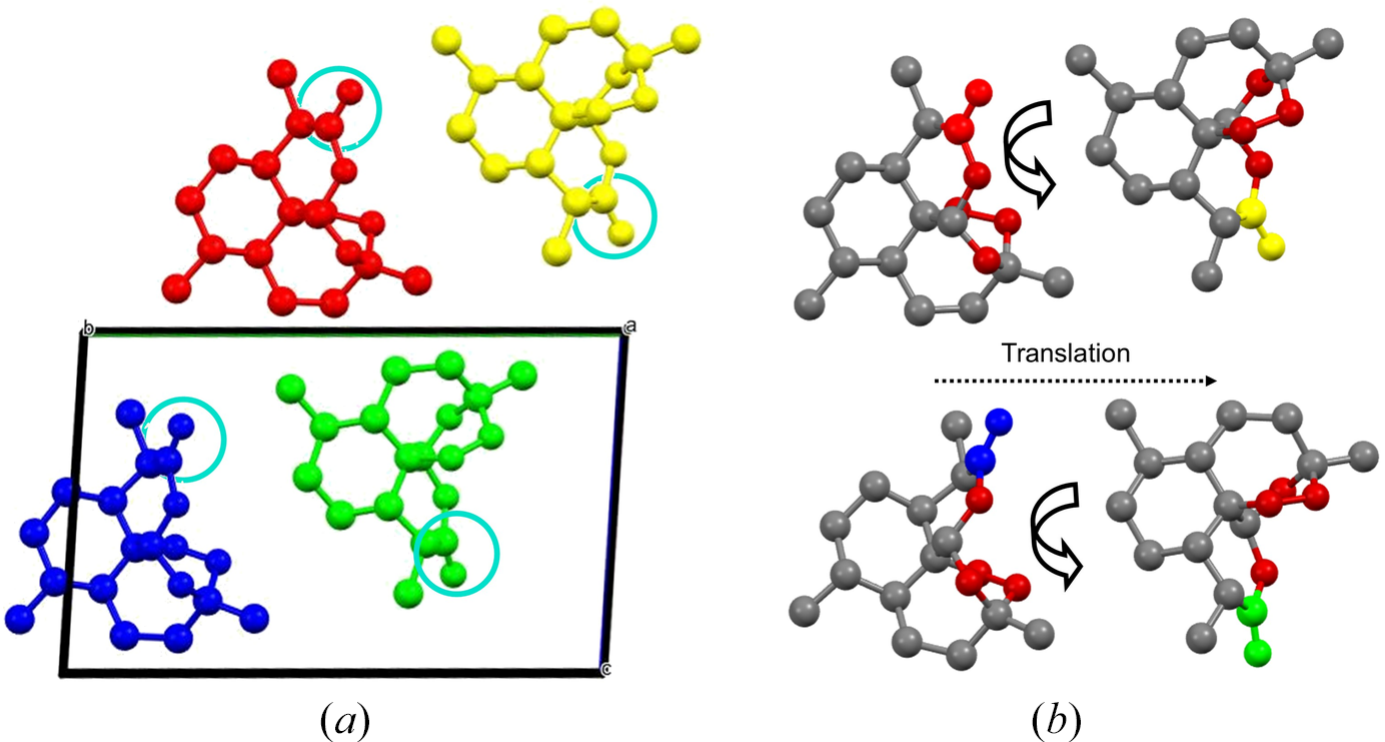
(*a*) Molecular arrangement of ART form (II) at ambient conditions showing the unit-cell contents (*Z* = 4) coloured by symmetry equivalence. Blue circles highlight similar carbonyl groups on each of the molecules that help to indicate the pseudo-2_1_ relationship between the molecules (red with yellow & blue with green). (*b*) The asymmetric unit coloured by element with exception of the carbonyl groups which are colour coded to link to Fig. 5 ▸(*a*). The curved arrows represent the pseudo twofold rotation where the red molecule rotates and translates (dotted line) to the yellow molecule forming the pseudo 2_1_-screw axis.

Chan *et al.* (1997 ▸) recrystallized ART from cyclo­hexane and identified the crystals as a new polymorph, form (II), which can be described by four molecules in the asymmetric unit. Form (II) manifests as thin, plate-like crystals, in contrast to form (I), which appears as thick rods. The density of form (II) is slightly lower than that of form (I), which is in line with the known stability and density rule of Burger & Ramberger (1979 ▸). Chan *et al.* (1997 ▸) highlighted the main difference between the polymorphs to be the different torsion angles, particularly around the ring systems where they can be up to 15° different. One of the key structural observations of form (II) is the relationship between the independent molecules. The molecules exhibit pseudo 2_1_-screw axis along the *b* direction for the two sets of molecules [Fig. 5 ▸(*a*)] relating the red to the yellow molecule, and blue molecule to green molecule. The molecular packing lacks a formal symmetry relationship, due to slight variations in the spatial arrangement of the molecules.

#### 3.2.1. Effect of pressure on ART form (II)

**Figure 6:**
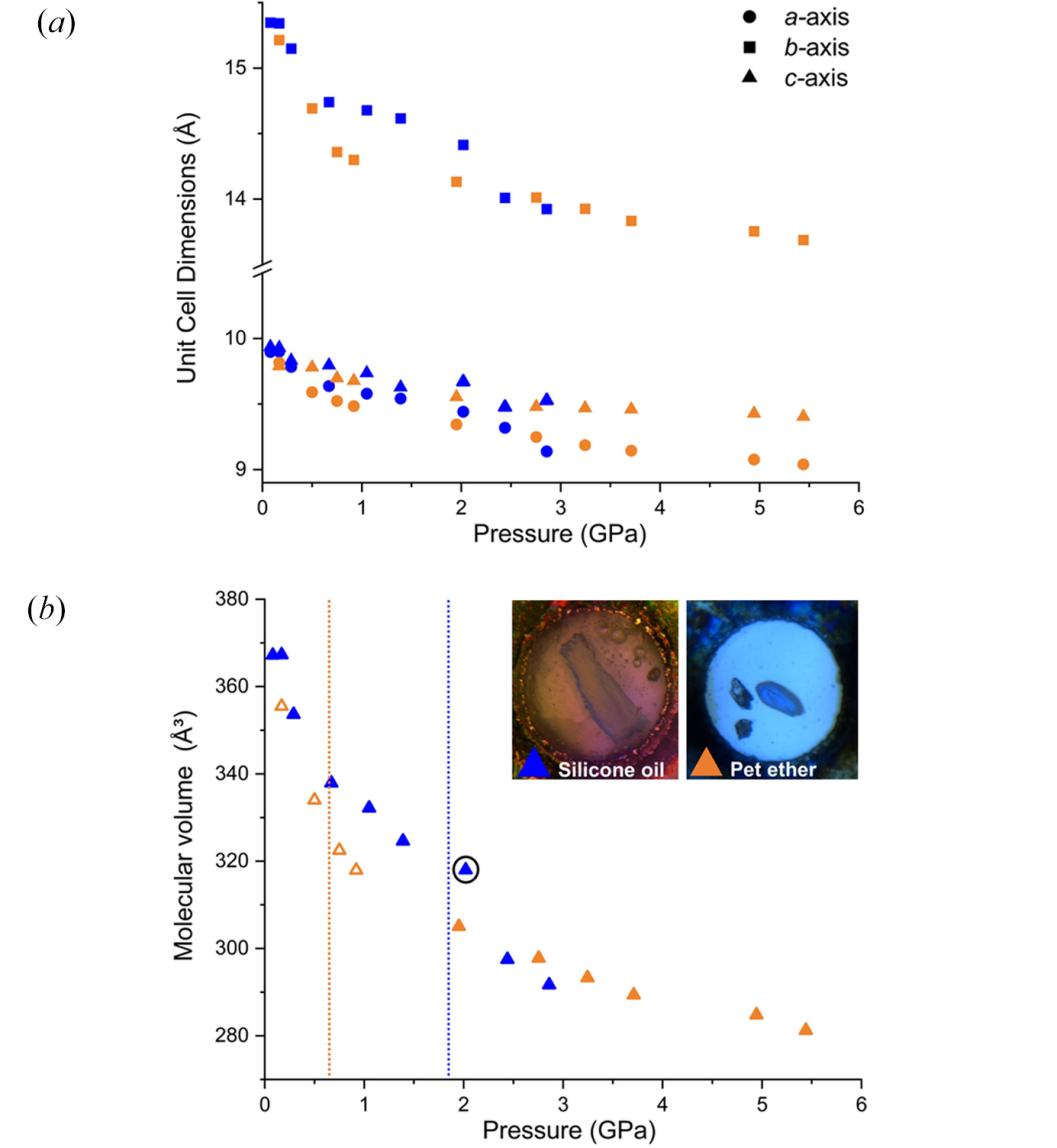
(*a*) Unit-cell dimensions of two different SC studies; blue shapes represent SC in silicone oil (crystal not annealed); orange shapes represent annealed SC compressed using petroleum ether as the PTM. (*b*) Molecular volume of ART for all studies. The blue triangles represent the study in silicone oil and the orange triangles represent the two studies in petroleum ether (filled: initial study; hollow: second study with unit cells only). Inset are pictures of the crystals used in the studies. The hole size of gasket is 250 µm for comparison of crystal size.

One of the key challenges with the study of form (II) was that the crystals are very thin and fragile. Many of the crystals that we tested in the DAC did not diffract strongly enough for data collection, hence the data on form (II) was collated from two different studies: one in petroleum ether and the other in silicone oil (Fig. 6 ▸). The initial study using petroleum ether as the PTM was conducted using an annealed single crystal from cyclo­hexane. The annealing process was performed in a DAC without measurable pressure applied but enough to seal the system. Due to the low freezing pressure of cyclo­hexane (0.025 GPa) (Würflinger, 1975 ▸), the crystal was removed from the DAC and reloaded into another DAC for the compression study. Unfortunately, the initial compression of this crystal was to 1.95 GPa. At this pressure, the crystal has transformed to a new polymorph; however, we continued to study this phase to a maximum pressure of 5.4 GPa before decompression. There was no characterization of the crystal after the initial annealing to verify the starting polymorph; hence, we could not definitively assign this change to the compression process or whether annealing caused the change in polymorph. Therefore, a further study was required to identify the source of phase transition. Fortunately, a single crystal of sufficient quality was obtained from the ambient solution crystallizations without the requirement to anneal it. On this occasion, the crystal was loaded using silicone oil due to the scarcity of crystals of sufficient size and quality. Petroleum ether is highly volatile and, in our experience, leads to a higher proportion of failed loadings, *i.e.* where liquid is not present in cell or where the crystal is washed away from the diamond tip.

The data collected from a crystal in silicone oil [Fig. 6(*b*) ▸, blue triangles] indicate a smooth compression of the structure to ∼2 GPa. From ambient pressure to 2 GPa, the *a* and *c* axes compress to a similar extent reducing by 4.1%. Above 2 GPa, the *b* axis exhibits a sharp decrease indicating a single crystal to single crystal phase transition from form (II) to a new high-pressure polymorph that will be designated form (III), confirming our initial observation in petroleum ether.

From both the silicone oil and petroleum ether studies, we have been able to observe that the new phase has unit-cell parameters similar to form (II) but is now described as a monoclinic *P*2_1_ structure (Table 3 ▸). The phase transition is characterized by a change from a *Z*′ = 4 structure to a *Z*′ = 2 structure through the increase in symmetry. The molecular volume of form (III) is 8.4% lower than form (II) indicating an increase in the density of the phase. Using void space analysis, form (II) exhibits a void space of 156.01 Å^3^, accounting for 12% of the unit-cell volume, whereas the new monoclinic phase shows a reduced void space of 128.40 Å^3^ or 10.1% of the unit cell. One of the unusual observations was at 2.02 GPa (indexing only), where there was a change in symmetry indicated by the indexing of a monoclinic unit cell, but not a significant change in molecular volume [Fig. 6 ▸(*b*); circled point]. This suggests that the change in symmetry and volume can be decoupled from one another in a two-stage process. Firstly, the molecules arrange themselves into the true *P*2_1_ symmetry before the volume fully compresses. The volume reduction associated with the new form occurs later at 2.44 GPa.

A further notable result from our examination of the two media was that the choice of PTM influences the pressure at which the phase transition occurs. For the petroleum ether study [orange triangles, Fig. 6 ▸(*b*)] multiple different crystals were used, and we discovered that the new phase can be obtained at pressure as low as 0.75 GPa using an indexed crystal (hollow orange triangles); these results are consistent across different crystals. To provide evidence as to the basis for this change, we observed that petroleum ether caused a visible shrinkage of the crystal indicating ART has some solubility in petroleum ether. This solubility may promote the transition to the new phase through solvent-mediated phase transition and nucleation on the surface of the mother crystal. This is not observed in silicone oil in which ART is insoluble, hence, the transition is delayed to higher pressure. It is not the first time PTM has been shown to impact the polymorphism of materials. Zakharov *et al.* (2016 ▸) demonstrated this in their study on β-chlorpropamide in various media (*e.g.* helium, paraffin). For example, when He was used as the PTM the phase transition from the orthorhombic β-polymorph to the new monoclinic phase, β^I^_HP_, appeared between 0.3 to 0.5 GPa. However, when using liquid paraffin as the PTM, unit-cell parameters similar to β^I^_HP_-chlorpropamide were observed at 0.1 GPa, and upon further compression to 0.3 GPa, a new triclinic phase, β^III^_HP_ was formed.

### 3.3. Compression of form (III)

**Figure 7:**
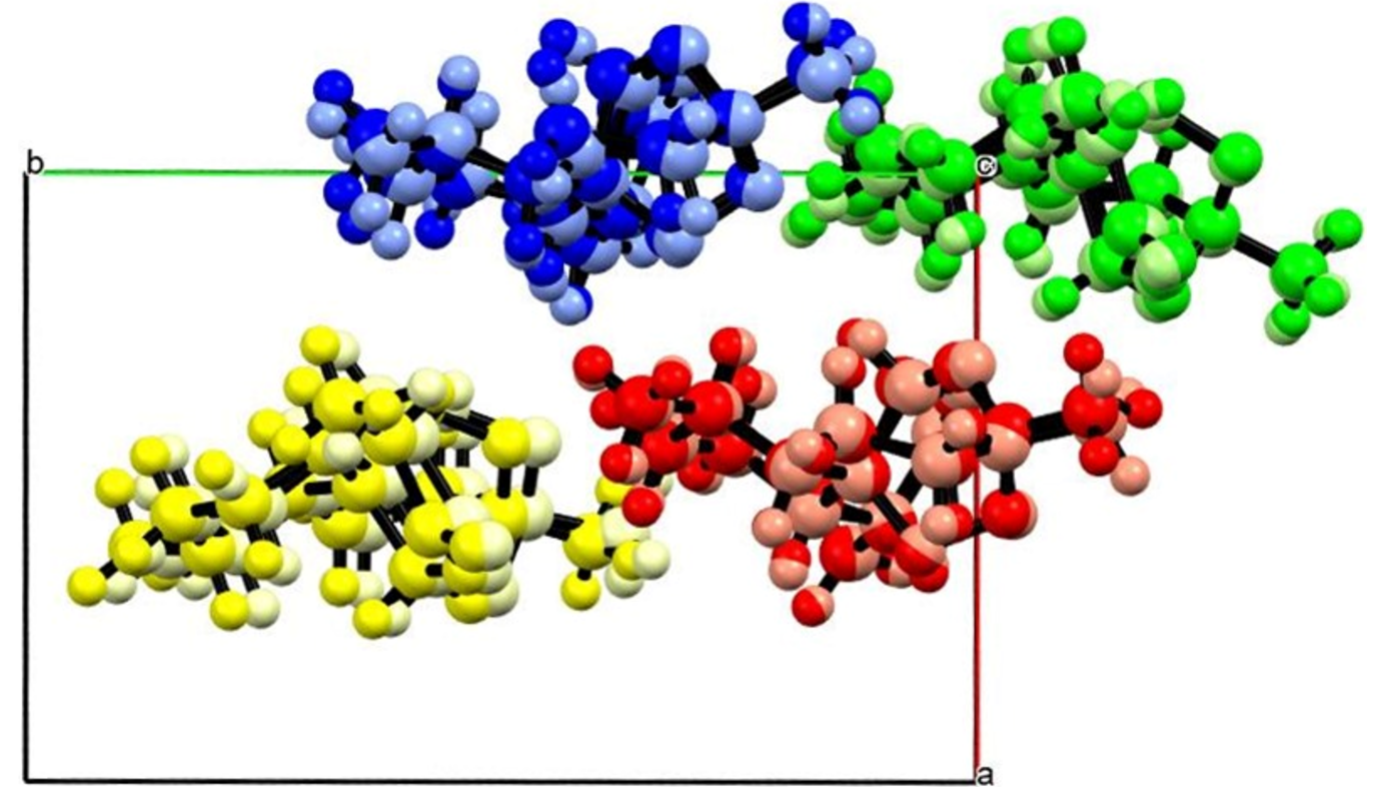
Overlay of the triclinic and high-pressure monoclinic forms of ART. The darker-coloured molecules represent the triclinic form at 1.39 GPa [highest form (II) data pressure], while the pastel-coloured molecules represent the high-pressure monoclinic form at 1.95 GPa. The root-mean-square deviation is 0.305 with 15/15 molecules overlapping indicating a strong similarity. The β angle remains consistent between the phases with the alpha and gamma becoming 90°. This is achieved through the chains sliding with respect to each other along the *b* direction.

The new high-pressure phase possesses a monoclinic *P*2_1_ structure with *Z*′ = 2. As mentioned previously, the molecules in form (II) are related by a pseudo-2_1_ axis along the *b* axis. Over the phase transition, form (III) adopts a true 2_1_ screw axis formalizing the relationship between the molecules. This leads to a reduction in the length of the *b* axis allowing for denser and more efficient molecular packing. A comparison the structures of forms (II) and (III) indicates a packing similarity of 15/15 molecules and a root-mean-square similarity score of 0.305 indicating a strong similarity which explains how the single crystal is maintained over the transition (Fig. 7 ▸). A comparison of the unit-cell parameters for forms (I), (II), and (III) at closer pressure points is found in Table 4 ▸.

**Figure 8:**
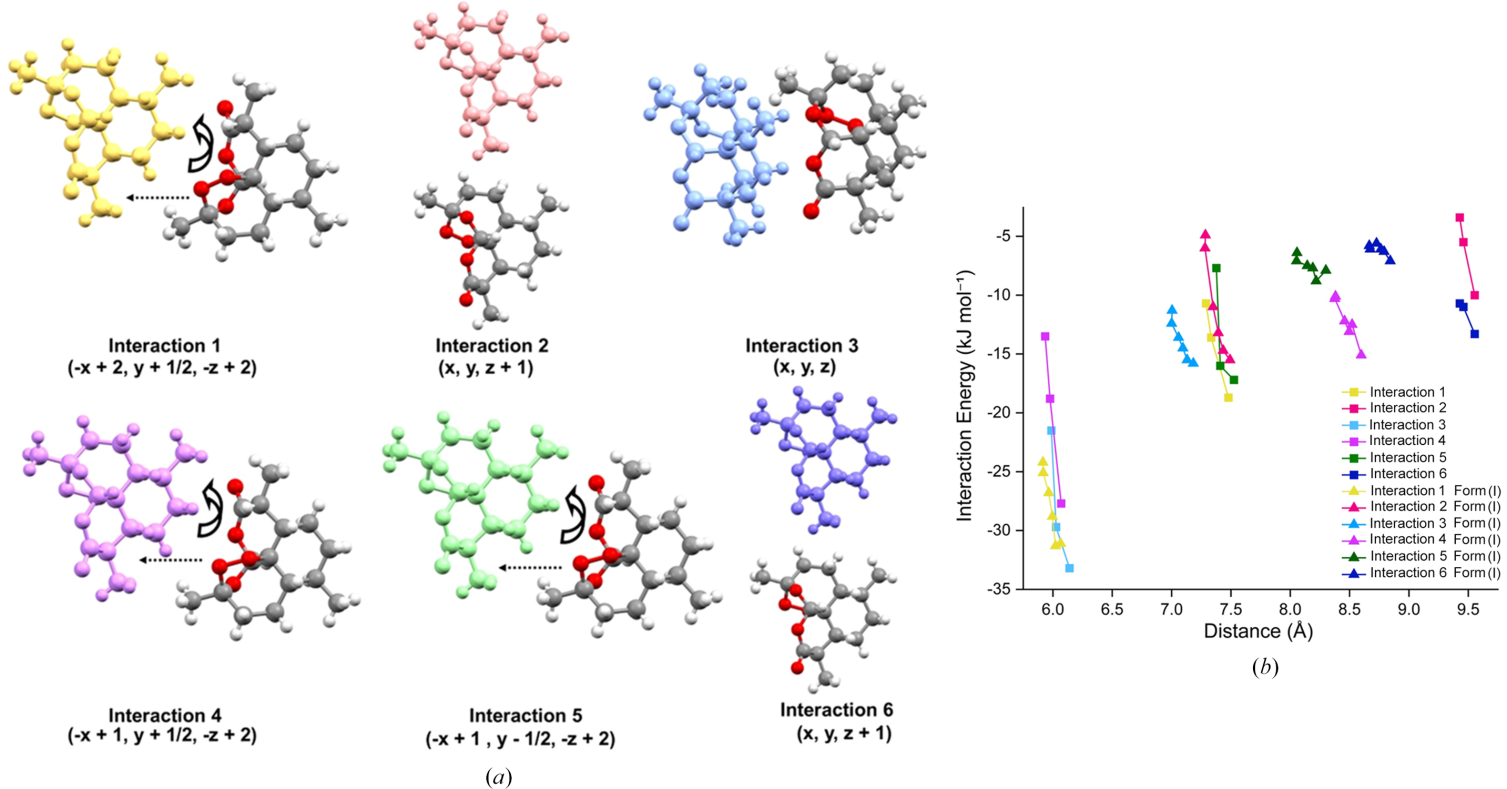
(*a*) Structures of the highest-energy interactions in the ART structure from PIXEL analysis. Molecules in grey represent the central molecule and the coloured molecule represents the transformed molecules. The curved arrows represent the pseudo-twofold rotation, and the dotted arrows highlight the translation in the pseudo 2_1_-screw axis. (*b*) Graph of the total interaction energy (in kJ mol^−1^) against the distance between the molecular centroids of the molecules involved in the interaction (in Å) for form (III) shown as square symbols. Triangles represent form (I) interactions over a similar pressure range. Lines serve as a guide to the eye.

Pixel analysis of form (III) has been performed on three pressure points from 1.95 GPa onwards. The molecules in interactions 1, 4 and 5 are related by a 2_1_ screw axis and these contacts extend along the *b* axis [Fig. 8 ▸(*a*)]. Interaction 3 (blue) and interaction 4 (purple) are the most stabilizing as the total energies reach approximately −30 kJ mol^−1^ at centroid distances near 6.0 Å. Interaction 3 displays a much steeper curve as the repulsive component increases significantly from 55.1 to 110.7 kJ mol^−1^ with pressure. A comparison of the interactions over a similar pressure range in form (III) [Fig. 8 ▸(*b*), square data points] and form (I) [Fig. 8 ▸(*b*), triangle data points] provides a clearer insight into how their packing responds differently under compression [Fig. 8 ▸(*b*)]. The response of the two forms to pressure is similar with all interactions showing an increase in energy due to the increase in repulsive forces. Form (III) shows a more dispersed set of interactions over the computed range, specifically, interactions 2 and 6 occurring at longer centroid distances compared with form (I). The longer interactions of form (I) (interactions 4 to 6) are more face-to-face interactions of the ring systems as opposed to the edge-to-edge interactions in form (III) (interactions 2 and 6).

### 3.4. Comparison of polymorphic forms of ART

The mechanical responses of the three polymorphic forms of ART were fitted to the BMEoS, revealing distinct differences in compressibility.Form (I), fitted with a third-order BMEoS, exhibits a low bulk modulus *K*_0_ of 2.9 (10) GPa and *V*_0_ = 1448.1 (10) Å^3^, indicating its relatively soft and compressible nature. Due to the paucity of data, form (II) was fit using a second-order BMEoS with a bulk modulus [*K*_0_] of 7.7 (11) GPa and *V*_0_ = 1480 (16) Å^3^. Form (III) is stiffer, with a bulk modulus of 12 (4) GPa and *V*_0_ = 1357 (18) Å^3^ also determined using a third-order BMEoS. The smaller *V*_0_ of form (III) points to a denser packing arrangement, consistent with its higher bulk modulus. The higher uncertainties are a due to a paucity of data at low pressures given the narrow stability range of form (III).

**Figure 9:**
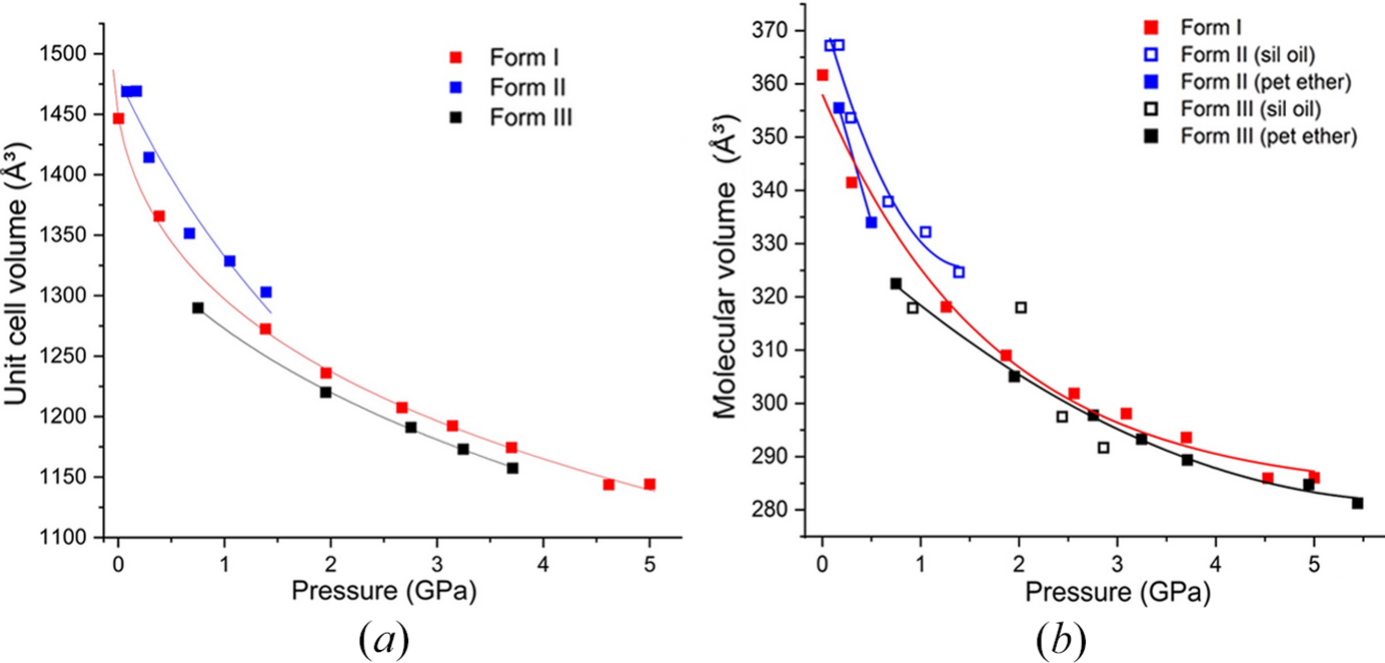
Unit-cell volume fitted with third-order BMEoS for forms (I) and (III), and second-order BMEoS for form (II). (*b*) Molecular volume versus pressure summary of all studies with different crystals or different PTM. Form (I) is represented by red squares; form (II) is in blue squares and form (III) is in black squares; closed squares represent petroleum (pet) ether studies; open squares are silicone (sil) oil studies.

The data shown in Fig. 9 ▸ represent molecular volume data from multiple experiments on different crystals of each form highlighted by polymorph. Data for form (II) were only collected to 1.39 GPa due to the phase transition and shows slightly higher molecular volumes than forms (I) and (III). Notably, the silicone oil studies (open blue squares) of form (II) show slightly higher molecular volume at similar pressures compared to petroleum ether studies [closed blue squares; Fig. 9 ▸(*b*)] suggesting a better transmission of pressure to the sample with this media over silicone oil. At higher pressures, forms (I) and (III) converge to a similar molecular volume at ∼5 GPa with a subtle difference in molecular volume at 5.4 GPa.

**Figure 10:**
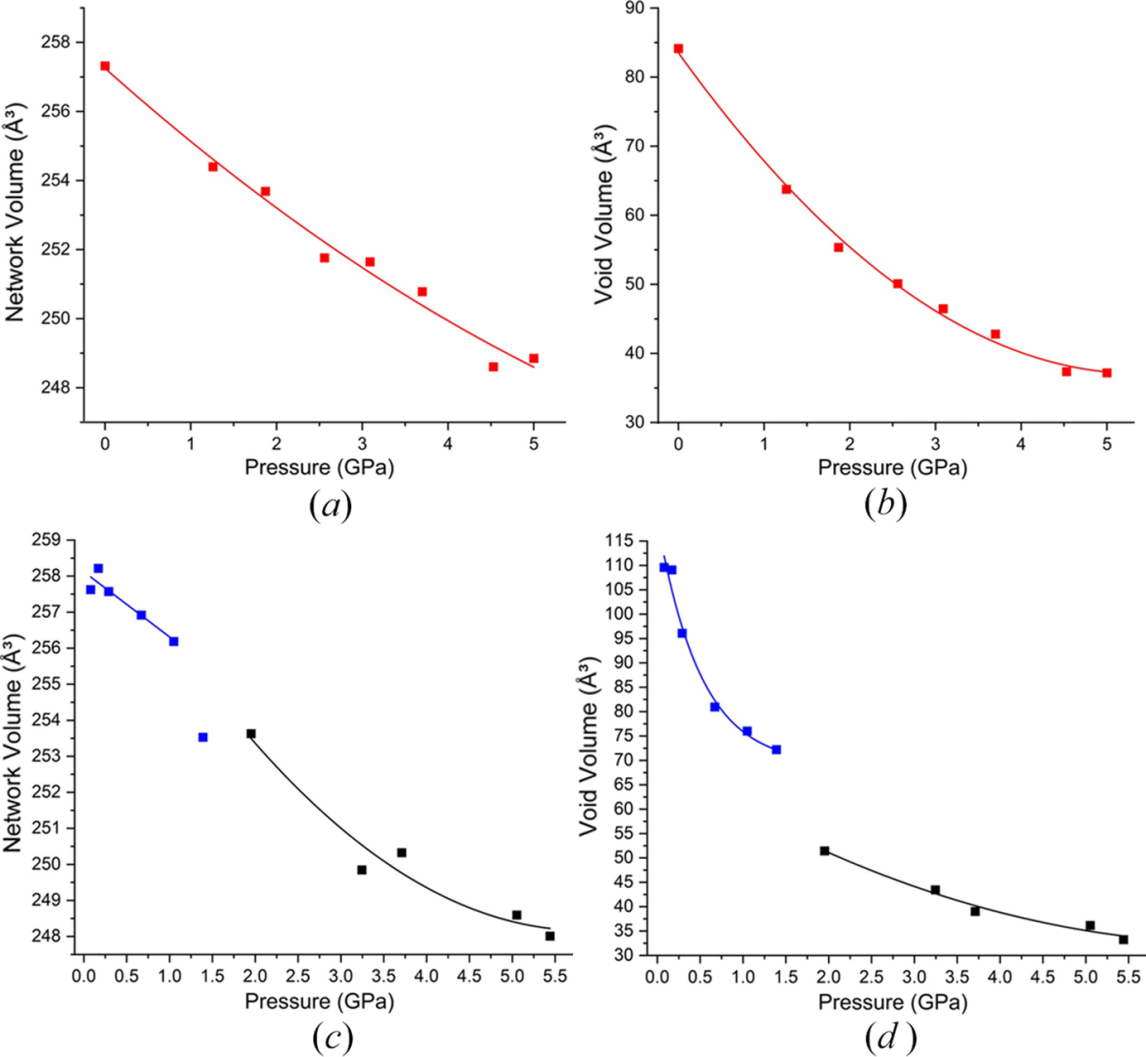
*CellVol* (Wilson *et al.*, 2022 ▸) analysis for ART (*a*) form (I) network volume against pressure (GPa) in red. (*b*) Void volume of form (I) against pressure. (*c*) Form (II) in silicone oil (blue) and form (III) in petroleum ether (black) network volume against pressure. (*d*) Form in silicone oil (blue) and form (III) in petroleum ether (black) void volume against pressure. The form (II) to form (III) phase transition is observed between 1.5 and 2.02 GPa in silicone oil. Lines serve as a guide to the eye.

Void spaces in crystal structures provide an opportunity to rationalize the behaviour of organic materials under pressure. Wilson *et al.* (2022 ▸) have proposed a method by which the volume of the crystal structure can be separated into two components: (i) the network volume including space occupied by atoms and their intermolecular contacts (based on van der Waals radii) and (ii) the void volume based on unoccupied space. This method addresses the issue of access to enclosed void spaces when the rolling ball method is employed. Using this method, we have analysed the changes in both the network and void volumes as a function of pressure (Fig. 10 ▸). Form (I) compresses smoothly across both the network and void volumes to 5 GPa without any distinct changes to the mechanism of compression. However, the compression of form (II) shows discontinuities in both the network and void volumes over the phase transition.

One of the key and unusual observations for form (II), from the molecular volume and symmetry perspective, was at 2.02 GPa where the structure seemed to change symmetry, yet the molecular volume remained consistent before reducing in the next data point. By deconvoluting the compression into network and void volumes we are able to see how the compression changes over this pressure range and provide further detail to enhance our understanding of the behaviour which we believe is a two-step process. Prior to the phase transition (between 1.05 and 1.39 GPa), the network volume shows a drop in volume which was coined by Wilson *et al.* (2022 ▸) as ‘premonitory behaviour’ in their explanation for the behaviour in 3-fluoro­salicylaldoxime. In their work, the authors used a Vinet third-order EoS to fit the network volume of 3-fluoro­salicylaldoxime. They observed that the last two data points, at 5.8 and 6.5 GPa, fell below the fitted curve prior to a reconstructive transition, above which no further data could be collected. We see this effect in the compression of form (II). The drop in network volume means that while the volume occupied by the atoms and their intermolecular contacts has reduced; no discontinuity is observed in the void volume at this point. This signifies that in this first stage the molecules are starting to pre-arrange into a more efficiently packed arrangement. If we take the ‘chains’ of molecules described in Fig. 5 ▸, *i.e.* red/yellow and blue/green pairs, we hypothesize that the molecules subtly reorganize so that they establish a relationship approximating a 2_1_-screw axis within each individual pair reducing the network volume prior to the transition. At the next pressure point, the symmetry is formalized with the chains sliding across each other (along the *b* direction) resulting in a structure that can be described in a monoclinic crystal system. The second stage is the increase in packing efficiency where the two chains compress together to reduce the void volume (29% reduction).

**Figure 11:**
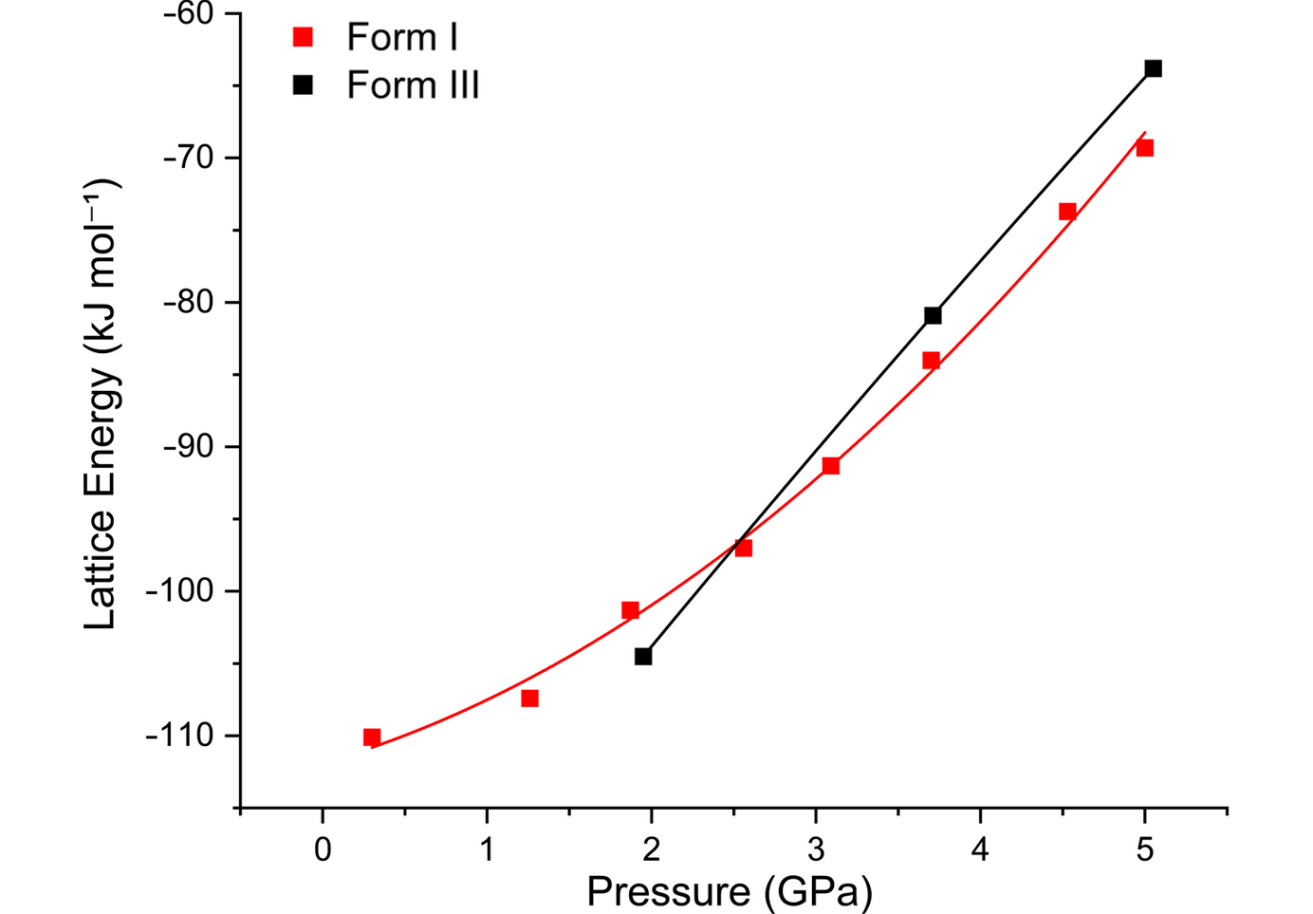
Lattice energy of form (I) (red squares) and form (III) (black squares) as a function of pressure.

Our investigation of the behaviour of ART polymorphs at high pressures shows clear variations in their phase stability and compressibility under different conditions. From an energetic perspective, the overall lattice energies of form (I) and form (III) indicate that they are close in energy at ∼1.9 GPa. Unfortunately, the high *Z*′ precludes the calculation of the lattice energy of form (II). Arguably, the rate of change of the lattice energy for form (III) is higher than for form (I) and we observe this in the overall repulsion energies where form (III) is 11.9 kJ mol^−1^ more repulsive over the same range [Fig. 11 ▸; repulsion energies form (I): 226.6 to 387.5 kJ mol^−1^; form (III) (from 1.9 GPa) 256.3 to 429.1 kJ mol^−1^].

## 4. Conclusion

In conclusion, the high-pressure behaviour of the three ART polymorphs reveals distinct structural responses to compression. Form (I) is the most compressible, suggesting a greater ability to react to external pressure. In contrast, form (II) undergoes a phase transition to a new high-pressure phase, form (III). Interestingly the transition is observed at 0.75 GPa in petroleum ether but delayed to 2.02 GPa in silicone oil, underscoring the importance of experimental conditions in polymorph stability and that the solubility in the PTM, however slight, can influence outcome. Form (III) is structurally related to form (II), where the molecules move from a pseudo-2_1_ screw axis in form (II) to a formal monoclinic *P*2_1_ structure, reducing the asymmetric unit from *Z*′ = 4 to *Z*′ = 2. Analysis of the transition shows that molecules pre-set themselves into a more efficient packing arrangement before the formal crystallographic symmetry is observed. This is a further observation that whilst the solid may exhibit the same solid-state structure, the molecular positions may yield information about how the molecules interact prior to any formal change in symmetry. This is particularly relevant in *Z*′ > 1 structures, where molecules can move independently from each other due to the lack of symmetry constraints.

## Supplementary Material

Crystal structure: contains datablock(s) FormI_1, FormI_2, FormI_3, FormI_4, FormI_5, FormI_6, FormI_7, FormI_8, FormII_1, FormII_2, FormII_3, FormII_4, FormII_5, FormII_6, FormIII_1, FormIII_2, FormIII_3, FormIII_4, FormIII_5, FormIII_6, FormIII_7, FormIII_8. DOI: 10.1107/S205252062600291X/aw5104sup1.cif

Structure factors: contains datablock(s) FormII_1. DOI: 10.1107/S205252062600291X/aw5104FormII_1sup2.hkl

Structure factors: contains datablock(s) FormII_2. DOI: 10.1107/S205252062600291X/aw5104FormII_2sup3.hkl

Structure factors: contains datablock(s) FormII_3. DOI: 10.1107/S205252062600291X/aw5104FormII_3sup4.hkl

Structure factors: contains datablock(s) FormII_4. DOI: 10.1107/S205252062600291X/aw5104FormII_4sup5.hkl

Structure factors: contains datablock(s) FormII_5. DOI: 10.1107/S205252062600291X/aw5104FormII_5sup6.hkl

Structure factors: contains datablock(s) FormII_6. DOI: 10.1107/S205252062600291X/aw5104FormII_6sup7.hkl

Structure factors: contains datablock(s) FormIII_1. DOI: 10.1107/S205252062600291X/aw5104FormIII_1sup8.hkl

Structure factors: contains datablock(s) FormIII_2. DOI: 10.1107/S205252062600291X/aw5104FormIII_2sup9.hkl

Structure factors: contains datablock(s) FormIII_3. DOI: 10.1107/S205252062600291X/aw5104FormIII_3sup10.hkl

Structure factors: contains datablock(s) FormIII_4. DOI: 10.1107/S205252062600291X/aw5104FormIII_4sup11.hkl

Structure factors: contains datablock(s) FormIII_5. DOI: 10.1107/S205252062600291X/aw5104FormIII_5sup12.hkl

Structure factors: contains datablock(s) FormIII_6. DOI: 10.1107/S205252062600291X/aw5104FormIII_6sup13.hkl

Structure factors: contains datablock(s) FormIII_7. DOI: 10.1107/S205252062600291X/aw5104FormIII_7sup14.hkl

Structure factors: contains datablock(s) FormIII_8. DOI: 10.1107/S205252062600291X/aw5104FormIII_8sup15.hkl

Structure factors: contains datablock(s) FormI_3. DOI: 10.1107/S205252062600291X/aw5104FormI_3sup16.hkl

Structure factors: contains datablock(s) FormI_4. DOI: 10.1107/S205252062600291X/aw5104FormI_4sup17.hkl

Structure factors: contains datablock(s) FormI_5. DOI: 10.1107/S205252062600291X/aw5104FormI_5sup18.hkl

Structure factors: contains datablock(s) FormI_6. DOI: 10.1107/S205252062600291X/aw5104FormI_6sup19.hkl

Structure factors: contains datablock(s) FormI_7. DOI: 10.1107/S205252062600291X/aw5104FormI_7sup20.hkl

Structure factors: contains datablock(s) FormI_8. DOI: 10.1107/S205252062600291X/aw5104FormI_8sup21.hkl

Structure factors: contains datablock(s) mo_artemisinin001_0m. DOI: 10.1107/S205252062600291X/aw5104FormI_1sup22.hkl

Structure factors: contains datablock(s) artemisinin003. DOI: 10.1107/S205252062600291X/aw5104FormI_2sup23.hkl

Includes Figs S1-S5 and Tables S1 and S2. DOI: 10.1107/S205252062600291X/aw5104sup24.pdf

Access to data once paper has been accepted and all data are added to the link: https://doi.org/10.15129/2af1df8c-307a-4478-bd47-82e5e5c32a4b

CCDC references: 2465849, 2465850, 2465851, 2465852, 2465853, 2465854, 2465855, 2465856

## Figures and Tables

**Table:** For all structures: C_15_H_22_O_5_, *M*_r_ = 282.32, orthorhombic, *P*2_1_2_1_2_1_, *Z* = 4, crystal size (mm) 0.25 × 0.18 × 0.09. Experiments were carried out at 296 K with Mo *K*α radiation using a Bruker APEX-II CCD. Absorption was corrected for by multi-scan methods (*SADABS*; Krause *et al.*, 2015 ▸). Refinement was on 184 parameters with 206 restraints. H-atom parameters were constrained.

	Form (I_1)	Form (I_2)	Form (I_3)	Form (I_4)
Crystal data				
Pressure (GPa)	0.3	1.26	1.87	2.56
PTM	Petroleum ether	Petroleum ether	Petroleum ether	Petroleum ether
*a*, *b*, *c* (Å)	6.2555 (4), 9.1513 (6), 23.860 (4)	6.1207 (4), 8.9193 (6), 23.311 (4)	6.0650 (4), 8.8429 (6), 23.048 (4)	6.0225 (4), 8.7906 (6), 22.807 (4)
*V* (Å^3^)	1365.9 (3)	1272.6 (2)	1236.1 (2)	1207.5 (3)
μ (mm^−1^)	0.10	0.11	0.11	0.12

Data collection				
*T*_min_, *T*_max_	0.667, 0.745	0.639, 0.745	0.644, 0.745	0.635, 0.745
No. of measured, independent and observed [*I* > 2σ(*I*)] reflections	5881, 748, 658	5140, 779, 665	5447, 747, 664	5183, 749, 652
*R*_int_	0.047	0.045	0.044	0.050
θ_max_ (°)	23.3	23.3	23.2	23.2
(sin θ/λ)max (Å^−1^)	0.556	0.555	0.555	0.555

Refinement				
*R*[*F*^2^ > 2σ(*F*^2^)], *wR*(*F*^2^), *S*	0.044, 0.122, 1.12	0.054, 0.182, 1.16	0.032, 0.071, 1.12	0.042, 0.116, 1.18
No. of reflections	748	779	747	749
Δρ_max_, Δρ_min_ (e Å^−3^)	0.14, −0.14	0.41, −0.36	0.12, −0.12	0.20, −0.22
Absolute structure	Flack *x* determined using 237 quotients [(*I*^+^) − (*I*^−^)]/[(*I*^+^) + (*I*^−^)] (Parsons *et al.*, 2013 ▸)	Flack *x* determined using 234 quotients [(*I*^+^) − (*I*^−^)]/[(*I*^+^) + (*I*^−^)] (Parsons *et al.*, 2013 ▸)	Flack *x* determined using 238 quotients [(*I*^+^) − (*I*^−^)]/[(*I*^+^) + (*I*^−^)] (Parsons *et al.*, 2013 ▸)	Flack *x* determined using 217 quotients [(*I*^+^) − (*I*^−^)]/[(*I*^+^) + (*I*^−^)] (Parsons *et al.*, 2013 ▸)
Absolute structure parameter	0.5 (8)	−0.1 (10)	−0.4 (9)	0.0 (10)

**Table:** 

	Form (I_5)	Form (I_6)	Form (I_7)	Form (I_8)
Crystal data				
Pressure (GPa)	3.09	3.7	4.53	5
PTM	Petroleum ether	Petroleum ether	Petroleum ether	Petroleum ether
*a*, *b*, *c* (Å)	5.9951 (3), 8.7630 (5), 22.698 (3)	5.9640 (4), 8.7274 (6), 22.562 (4)	5.9177 (3), 8.6735 (5), 22.285 (4)	5.9146 (5), 8.6661 (8), 22.322 (5)
*V* (Å^3^)	1192.44 (19)	1174.4 (2)	1143.8 (2)	1144.2 (3)
μ (mm^−1^)	0.12	0.12	0.12	0.12

Data collection				
*T*_min_, *T*_max_	0.6602, 0.7449	0.654, 0.745	0.654, 0.745	0.659, 0.745
No. of measured, independent and observed [*I* > 2σ(*I*)] reflections	5443, 739, 669	5226, 727, 643	5297, 575, 536	5001, 615, 549
*R*_int_	0.044	0.045	0.040	0.050
θ_max_ (°)	23.3	23.2	23.2	23.2
(sin θ/λ)max (Å^−1^)	0.555	0.555	0.553	0.554

Refinement				
*R*[*F*^2^ > 2σ(*F*^2^)], *wR*(*F*^2^), *S*	0.031, 0.070, 1.12	0.031, 0.070, 1.15	0.027, 0.064, 1.17	0.030, 0.071, 1.15
No. of reflections	739	727	575	615
Δρ_max_, Δρ_min_ (e Å^−3^)	0.09, −0.15	0.11, −0.14	0.08, −0.11	0.09, −0.13
Absolute structure	Flack *x* determined using 245 quotients [(*I*^+^) − (*I*^−^)]/[(*I*^+^) + (*I*^−^)] (Parsons *et al.*, 2013 ▸)	Flack *x* determined using 232 quotients [(*I*^+^) − (*I*^−^)]/[(*I*^+^) + (*I*^−^)] (Parsons *et al.*, 2013 ▸)	Flack *x* determined using 202 quotients [(*I*^+^) − (*I*^−^)]/[(*I*^+^) + (*I*^−^)] (Parsons *et al.*, 2013 ▸)	Flack *x* determined using 195 quotients [(*I*^+^) − (*I*^−^)]/[(*I*^+^) + (*I*^−^)] (Parsons *et al.*, 2013 ▸)
Absolute structure parameter	0.0 (9)	−1.6 (10)	−0.5 (9)	−0.6 (10)

† Computer programs: *SAINT* (Bruker, 2016 ▸), *SHELXT* (Sheldrick, 2015*b* ▸), *SHELXL* (Sheldrick, 2015*a* ▸), *Olex2* (Dolomanov *et al.*, 2009 ▸).

**Table:** 

	Form (II_1)	Form (II_2)	Form (II_3)	Form (II_4)
Crystal data				
Pressure (GPa)	0.08	0.17	0.29	0.67
PTM	Silicone oil	Silicone oil	Silicone oil	Silicone oil
*a*, *b*, *c* (Å)	9.898 (2), 15.347 (4), 9.93 (5)	9.900 (2), 15.342 (4), 9.930 (5)	9.7831 (12), 15.150 (2), 9.832 (3)	9.637 (2), 14.741 (4), 9.796 (5)
α, β, γ (°)	86.79 (4), 102.83 (4), 89.075 (14)	86.75 (4), 102.56 (4), 89.144 (14)	86.565 (19), 103.35 (19), 89.287 (8)	88.93 (3), 103.73 (3), 89.846 (14)
*V* (Å^3^)	1468.8 (9)	1469.2 (9)	1414.5 (5)	1351.6 (9)
μ (mm^−1^)	0.10	0.10	0.10	0.10

Data collection				
*T*_min_, *T*_max_	0.634, 0.745	0.614, 0.745	0.635, 0.745	0.647, 0.745
No. of measured, independent and observed [*I* > 2σ(*I*)] reflections	6845, 2686, 1019	7939, 2792, 1002	7362, 2713, 1129	5569, 2459, 1088
*R*_int_	0.073	0.086	0.073	0.070
θ_max_ (°)	23.4	23.4	23.3	23.2
(sin θ/λ)max (Å^−1^)	0.558	0.558	0.556	0.555

Refinement				
*R*[*F*^2^ > 2σ(*F*^2^)], *wR*(*F*^2^), *S*	0.067, 0.166, 0.96	0.068, 0.153, 0.98	0.062, 0.138, 0.97	0.075, 0.209, 0.98
No. of reflections	2686	2792	2713	2459
Δρ_max_, Δρ_min_ (e Å^−3^)	0.10, −0.12	0.11, −0.11	0.11, −0.15	0.14, −0.17
Absolute structure	Flack *x* determined using 333 quotients [(*I*^+^) − (*I*^−^)]/[(*I*^+^) + (*I*^−^)] (Parsons *et al.*, 2013 ▸)	Flack *x* determined using 333 quotients [(*I*^+^) − (*I*^−^)]/[(*I*^+^) + (*I*^−^)] (Parsons *et al.*, 2013 ▸)	Flack *x* determined using 372 quotients [(*I*^+^) − (*I*^−^)]/[(*I*^+^) + (*I*^−^)] (Parsons *et al.*, 2013 ▸)	Flack *x* determined using 344 quotients [(*I*^+^) − (*I*^−^)]/[(*I*^+^) + (*I*^−^)] (Parsons *et al.*, 2013 ▸)
Absolute structure parameter	1.0 (10)	1.1 (10)	1.6 (10)	−0.5 (10)

**Table:** 

	Form (II_5)	Form (II_6)
Crystal data		
Pressure (GPa)	1.05	1.39
PTM	Silicone oil	Silicone oil
*a*, *b*, *c* (Å)	9.579 (3), 14.679 (5), 9.737 (7)	9.542 (5), 14.616 (8), 9.628 (12)
α, β, γ (°)	88.89 (5), 103.90 (4), 89.854 (18)	88.97 (8), 103.94 (8), 89.81 (3)
*V* (Å^3^)	1328.7 (11)	1303 (2)
μ (mm^−1^)	0.11	0.11

Data collection		
*T*_min_, *T*_max_	0.618, 0.745	0.549, 0.745
No. of measured, independent and observed [*I* > 2σ(*I*)] reflections	3350, 1887, 888	2249, 1462, 590
*R*_int_	0.059	0.069
θ_max_ (°)	23.3	23.2
(sin θ/λ)max (Å^−1^)	0.555	0.555

Refinement		
*R*[*F*^2^ > 2σ(*F*^2^)], *wR*(*F*^2^), *S*	0.080, 0.227, 1.03	0.108, 0.351, 1.05
No. of reflections	1887	1462
Δρ_max_, Δρ_min_ (e Å^−3^)	0.18, −0.16	0.23, −0.24
Absolute structure	Flack *x* determined using 287 quotients [(*I*^+^) − (*I*^−^)]/[(*I*^+^) + (*I*^−^)] (Parsons *et al.*, 2013 ▸)	Flack *x* determined using 169 quotients [(*I*^+^) − (*I*^−^)]/[(*I*^+^) + (*I*^−^)] (Parsons *et al.*, 2013 ▸)
Absolute structure parameter	−1.0 (10)	−4.5 (10)

† Computer programs: *SAINT* (Bruker, 2016 ▸), *SHELXT* (Sheldrick, 2015*b* ▸), *SHELXL* (Sheldrick, 2015*a* ▸), *Olex2* (Dolomanov *et al.*, 2009 ▸).

**Table:** For all structures: C_15_H_22_O_5_, *M*_r_ = 282.32, monoclinic, *P*2_1_, *Z* = 4, crystal size (mm) 0.2 × 0.1 × 0.09. Experiments were carried out with Mo *K*α radiation using a Bruker D8 Venture diffractometer. Absorption was corrected for by multi-scan methods (*SADABS2016*; Krause *et al.*, 2015 ▸). Refinement was on 167 parameters with 47 restraints. H-atom parameters were constrained.

	Form (III_1)	Form (III_2)	Form (III_3)	Form (III_4)
Crystal data				
Pressure (GPa)	1.95	3.25	3.71	5.05
PTM	Petroleum ether	Petroleum ether	Petroleum ether	Petroleum ether
Temperature (K)	295	296	296	296
*a*, *b*, *c* (Å)	9.344 (2), 14.133 (3), 9.554 (5)	9.186 (3), 13.927 (5), 9.469 (8)	9.1432 (14), 13.835 (2), 9.459 (4)	9.076 (2), 13.755 (4), 9.428 (7)
β (°)	104.73 (4)	104.43 (6)	104.69 (3)	104.60 (4)
*V* (Å^3^)	1220.2 (8)	1173.1 (12)	1157.5 (6)	1139.1 (9)
μ (mm^−1^)	0.11	0.12	0.12	0.12

Data collection				
*T*_min_, *T*_max_	0.491, 0.745	0.384, 0.745	0.649, 0.745	0.358, 0.745
No. of measured, independent and observed [*I* > 2σ(*I*)] reflections	6348, 1335, 630	5153, 1256, 440	5284, 1210, 651	6090, 1265, 439
*R*_int_	0.150	0.276	0.108	0.328
θ_max_ (°)	23.5	23.4	23.3	23.4
(sin θ/λ)max (Å^−1^)	0.560	0.558	0.557	0.558

Refinement				
*R*[*F*^2^ > 2σ(*F*^2^)], *wR*(*F*^2^), *S*	0.074, 0.252, 0.86	0.154, 0.441, 1.06	0.063, 0.123, 1.00	0.151, 0.286, 1.01
No. of reflections	1335	1256	1210	1265
Δρ_max_, Δρ_min_ (e Å^−3^)	0.19, −0.23	0.52, −0.48	0.20, −0.17	0.24, −0.34
Absolute structure	Flack *x* determined using 213 quotients [(*I*^+^) − (*I*^−^)]/[(*I*^+^) + (*I*^−^)] (Parsons *et al.*, 2013 ▸)	Flack *x* determined using 93 quotients [(*I*^+^)−(*I*^−^)]/[(*I*^+^) + (*I*^−^)] (Parsons *et al.*, 2013 ▸)	Flack *x* determined using 222 quotients [(*I*^+^)−(*I*^−^)]/[(*I*^+^) + (*I*^−^)] (Parsons *et al.*, 2013 ▸)	Flack *x* determined using 119 quotients [(*I*^+^) − (*I*^−^)]/[(*I*^+^) + (*I*^−^)] (Parsons *et al.*, 2013 ▸)
Absolute structure parameter	0.5 (10)	−4.9 (10)	−0.3 (10)	−1.8 (10)

**Table:** 

	Form (III_5)	Form (III_6)	Form (III_7)	Form (III_8)
Crystal data				
Pressure (GPa)	5.44	4.52	3.88	2.57
PTM	Petroleum ether	Petroleum ether	Petroleum ether	Petroleum ether
Temperature (K)	296	296	296	296
*a*, *b*, *c* (Å)	9.0393 (13), 13.688 (2), 9.404 (4)	9.1279 (16), 13.819 (3), 9.461 (4)	9.1812 (15), 13.865 (3), 9.479 (4)	9.3601 (11), 14.1006 (18), 9.587 (3)
β (°)	104.79 (3)	104.86 (3)	104.89 (3)	104.87 (2)
*V* (Å^3^)	1125.0 (5)	1153.5 (6)	1166.2 (6)	1222.9 (5)
μ (mm^−1^)	0.12	0.12	0.12	0.11

Data collection				
*T*_min_, *T*_max_	0.580, 0.745	0.658, 0.745	0.392, 0.745	0.659, 0.745
No. of measured, independent and observed [*I* > 2σ(*I*)] reflections	5919, 1239, 621	6247, 1259, 606	5516, 1332, 584	6478, 1324, 644
*R*_int_	0.133	0.137	0.168	0.120
θ_max_ (°)	23.4	23.3	23.3	23.3
(sin θ/λ)max (Å^−1^)	0.559	0.557	0.557	0.556

Refinement				
R[F^2^ > 2σ(F^2^)], *wR*(F^2^), S	0.064, 0.140, 0.98	0.062, 0.129, 0.99	0.092, 0.256, 0.97	0.066, 0.149, 0.98
No. of reflections	1239	1259	1332	1324
Δρ_max_, Δρ_min_ (e Å^−3^)	0.18, −0.18	0.18, −0.20	0.25, −0.30	0.16, −0.15
Absolute structure	Flack *x* determined using 213 quotients [(*I*^+^) − (*I*^−^)]/[(*I*^+^) + (*I*^−^)] (Parsons *et al.*, 2013 ▸)	Flack *x* determined using 206 quotients [(*I*^+^) − (*I*^−^)]/[(*I*^+^) + (*I*^−^)] (Parsons *et al.*, 2013 ▸)	Flack *x* determined using 182 quotients [(*I*^+^) − (*I*^−^)]/[(*I*^+^) + (*I*^−^)] (Parsons *et al.*, 2013 ▸)	Flack *x* determined using 213 quotients [(*I*^+^) − (*I*^−^)]/[(*I*^+^) + (*I*^−^)] (Parsons *et al.*, 2013 ▸)
Absolute structure parameter	0.9 (10)	4.4 (10)	−1.1 (10)	−1.4 (10)

† Computer programs: *SAINT* (Bruker, 2016 ▸), *SHELXT* (Sheldrick, 2015*b* ▸), *SHELXL* (Sheldrick, 2015*a* ▸), *Olex2* (Dolomanov *et al.*, 2009 ▸).

**Table 4:** Structural parameters of ART polymorphs at closest pressure points

Form	Form (I)	Form (II)	Form (III)
Pressure (GPa)	0.30 (5)†	0.29 (5)	0.75 (5)
*a* (Å)	6.2555 (4)	9.7831 (12)	9.523 (5)
*b* (Å)	9.1513 (6)	15.150 (2)	14.360 (8)
*c* (Å)	23.860 (4)	9.832 (3)	9.697 (15)
α (°)	90	86.565 (19)	90
β (°)	90	103.385 (19)	103.30 (9)
γ (°)	90	89.287 (8)	90
*V* (Å^3^)	1365.9 (3)	1414.5 (5)	1290 (2)
Space group	*P*2_1_2_1_2_1_	*P*1	*P*2_1_
*Z*	4	4	4
*Z*′	1	4	2

† Standard uncertainty of ±0.05 GPa (Chervin *et al.*, 2001 ▸).

## Data Availability

The authors would like to acknowledge that all data underpinning this publication are openly available from Zenodo repository(doi: https://doi.org/10.5281/zenodo.19714537).
