# Supplementary material for: Form (III) of artemisinin: discovery and crystallographic characterization of a new high-pressure polymorph
Source: Acta Crystallogr B Struct Sci Cryst Eng Mater. 2026 May 11;82(Pt 3):316–29. doi: 10.1107/S205252062600291X (PMC13238485; doi:10.1107/S205252062600291X)
Supplement: Supplementary file 24 [file b-82-00316-sup24.pdf]

STRUCTURAL SCIENCE
CRYSTAL ENGINEERING
MATERIALS

Volume 82 (2026)

Supporting information for article:

Form III of Artemisinin: Discovery and crystallographic characterisation of a new high-pressure polymorph

Banaz Fetah, Lauren E Connor, Cheryl L Doherty and Iain DH Oswald

Form III of Artemisinin: Discovery and crystallographic characterisation of a new high-pressure polymorph

Banaz Fetah[a], Lauren E Connor[bc], Cheryl L Doherty[d] and Iain DH Oswald[a]*

[a]Strathclyde Institute of Pharmacy & Biomedical Sciences (SIPBS), University of Strathclyde, 161 Cathedral Street, Glasgow, G4 0RE G4, United Kingdom

[b] University of Strathclyde, 161 Cathedral Street, Glasgow, G4 0RE, United Kingdom

[c]Biomedical Research, Novartis, Basel, 4002, Switzerland

[d]Material Science, GlaxoSmithKline (United Kingdom), Gunnels Wood Rd, Stevenage, SG1 2NY, United Kingdom

Correspondence email: iain.oswald@strath.ac.uk

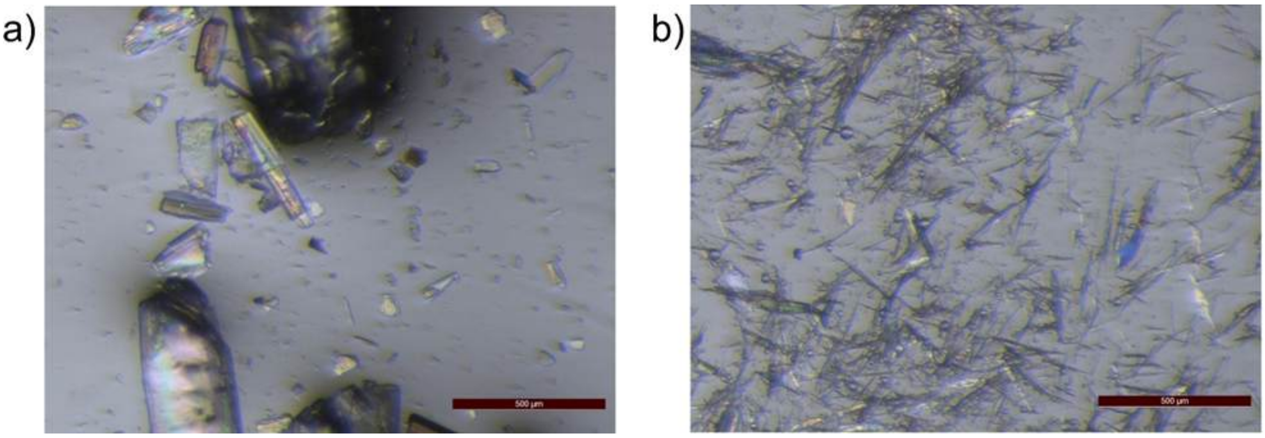

Figure S1 a) Orthorhombic Form I crystals b) Triclinic Form II crystals. The scale bars represent 500 μm

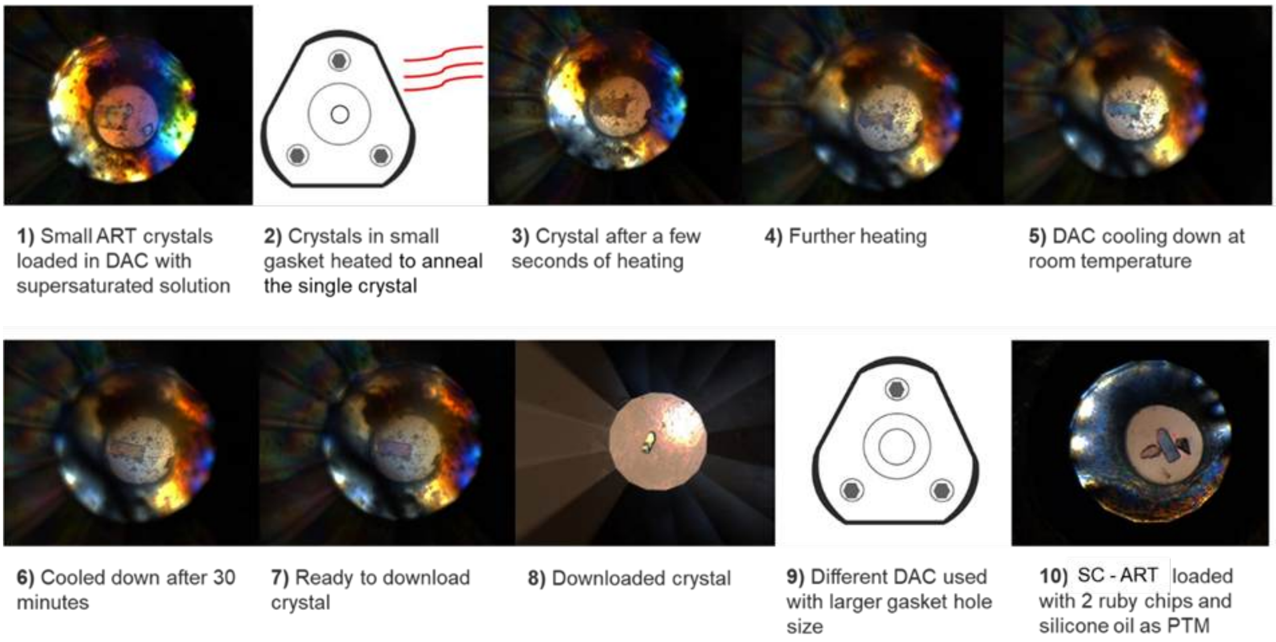

Figure S2 a) Annealing process of ART Form II crystals using a supersaturated solution of cyclohexane. The gasket (250 μm thickness) was indented to ~100 μm before a hole was drilled.

<u>Compression of Artemisinin</u>

In one study, the pressure was increased to 1.95 GPa due to the crystal being displaced on loading. Pressure was increased until the crystal remained static. Despite the increased pressure, the data quality remained acceptable. The pressure of the cell was increased to a maximum pressure of 5.44 GPa before decompression (Figure S3). The compression data shows a continuous decrease across all three axes with increasing pressure to 3.71 GPa where there seems to be an abrupt change in the compression behaviour between 3.71 and 5 GPa which might suggest a potential new phase transition. The new high-pressure phase is retained upon decompression. The unit cell lengths elongate but this path does not retrace the compression path, indicating some hysteresis of the sample.

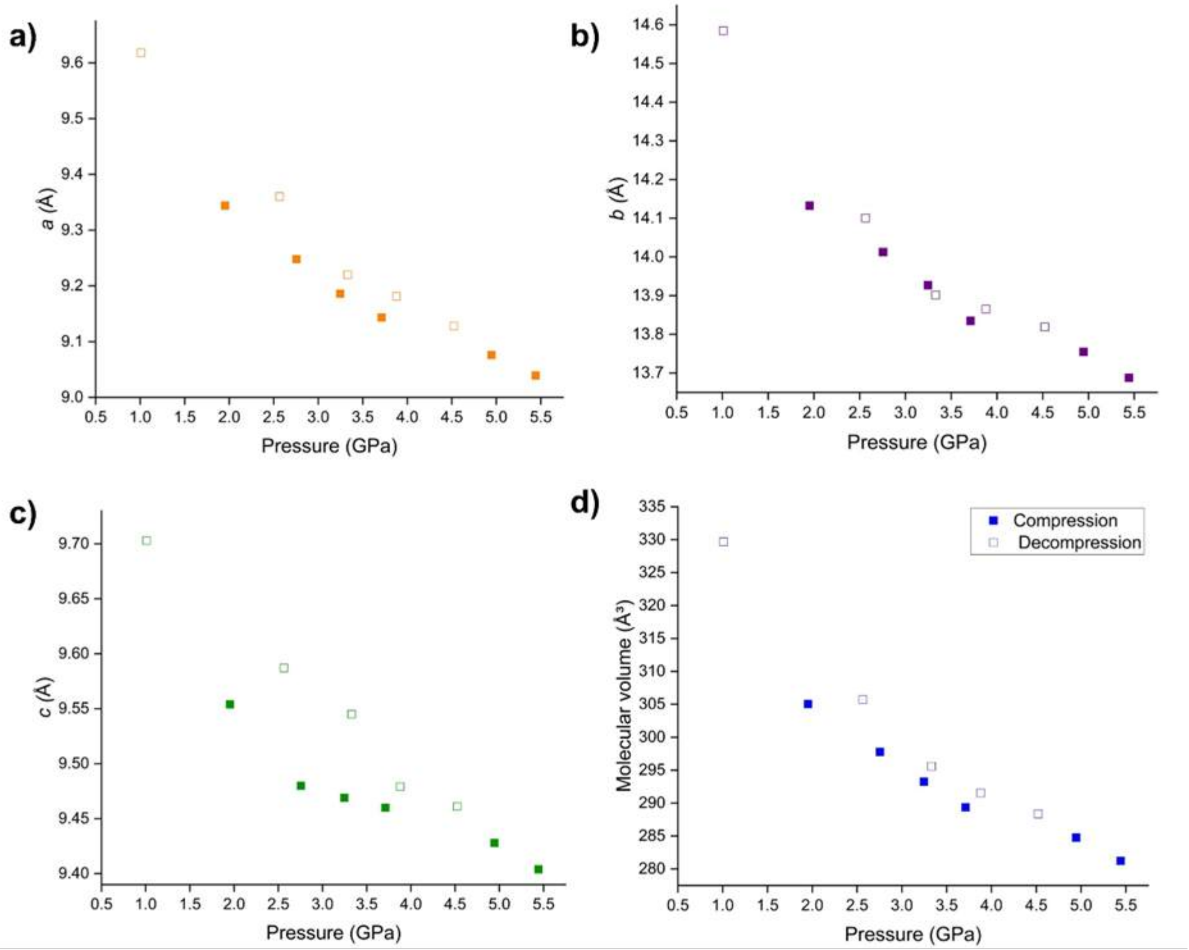

Figure S3 a) *a* axis (Å) of Artemisinin as a function of pressure (GPa) b) *b* axis (Å) of Artemisinin as a function of pressure (GPa) c) *c* axis (Å) of Artemisinin as a function of pressure (GPa) d) Molecular volume (Å^3) of Artemisinin as a function of pressure (GPa)

The crack on the crystal of Form II (Figure S4)) did not affect our ability to access good high-pressure data.

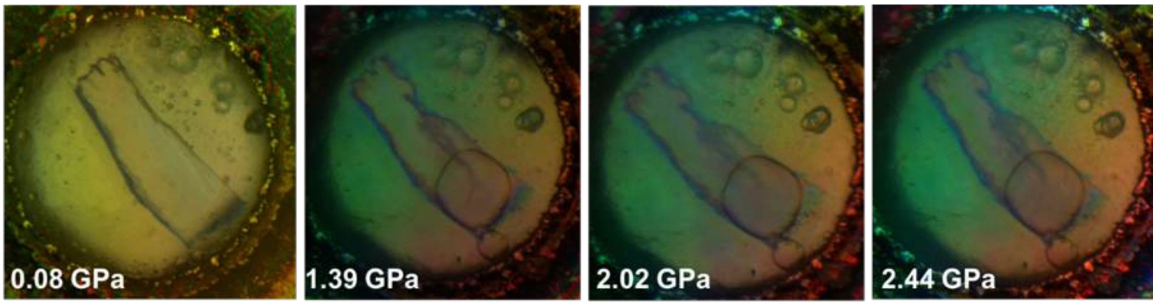

Figure S4 a) SC-ART loaded in silicone oil with ruby. SC obtained through evaporation technique from previous batch

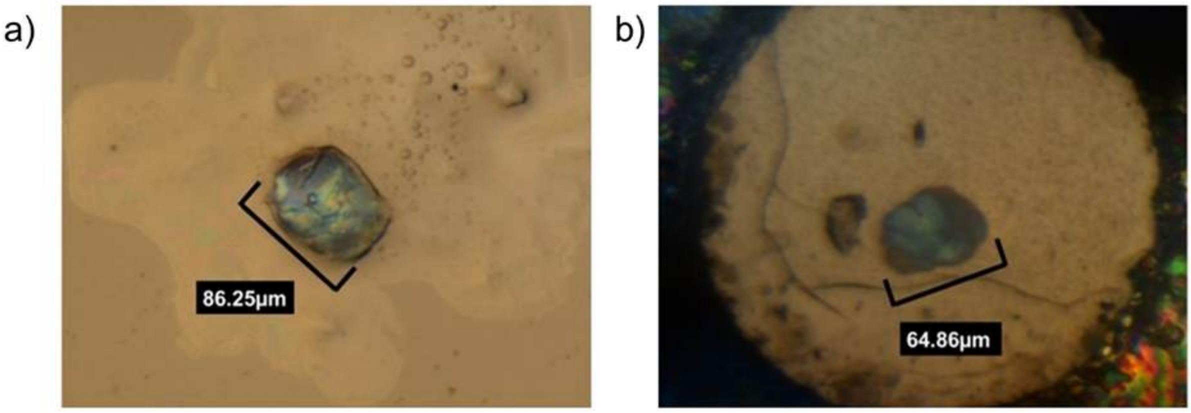

Figure S5 Crystal behaviour a) Crystal before adding petroleum ether b) Crystal shrinking after adding pet ether at ambient pressure

Table S1 Distance (Å), lattice energies and total energy at various pressures (GPa) for Artemisinin

Pressure	Centroid–centroid distance (Å)	E ELEC (kJ mol−1)	E POL (kJ mol−1)	E DISP (kJ mol−1)	E REP (kJ mol−1)	E TOT (kJ mol−1)
0	6.255	-17.3	-9.6	-37.3	30.7	-33.5
1.26	6.121	-27	-13.8	-46.6	52.1	-35.4
1.87	6.065	-29.4	-14.8	-49.1	62.2	-31.1
2.56	6.022	-34.4	-17.8	-53.2	74.2	-31.3
3.09	5.995	-36.1	-17.9	-54.5	79.6	-28.8
3.7	5.964	-39.1	-19.4	-56.5	88.3	-26.8
4.53	5.918	-45.7	-22.7	-60.8	104.1	-25.1
5	5.915	-45.8	-22.7	-60.3	104.5	-24.2

Table S2 Increasing pressure (GPa) of Artemisinin with total energies of the 6 strongest interactions (energies in kJ mol^{-1})

Pressure	0	1.26	1.87	2.56	3.09	3.7	4.53	5
Interaction1	-33.5	-35.4	-31.1	-31.3	-28.8	-26.8	-25.1	-24.2
Interaction 2	-22.4	-16.1	-15.5	-14.7	-13.2	-11	-6	-4.9
Interaction 3	-15.8	-17.1	-15.8	-15.5	-14.5	-13.6	-12.4	-11.3
Interaction 4	-15.6	-14.2	-15.1	-12.5	-13.1	-12.2	-10.3	-10.1
Interaction 5	-8.3	-8.6	-7.9	-8.8	-7.7	-7.5	-7.1	-6.4
Interaction 6	-6.2	-6.8	-7.1	-6.3	-6.1	-5.6	-6.1	-5.8
